# Sexual Norms, Religiosity, and Stigma in the Epidemiology of HIV and STI: A Cross-National Ecological Study of 68 Countries

**DOI:** 10.3390/healthcare14213557

**Published:** 2026-10-22

**Authors:** Salim Yılmaz, Ahmet Can Küçükkurt, Birol Yetim, Yusuf Çelik

**Affiliations:** 1 Department of Health Management, Graduate School of Health Sciences, Acibadem Mehmet Ali Aydinlar University, Istanbul 34752, Türkiye; salim.yilmaz@acibadem.edu.tr (S.Y.); yusuf.celik@acibadem.edu.tr (Y.Ç.); 2 Department of Health Management, Faculty of Health Sciences, Acibadem Mehmet Ali Aydinlar University, Istanbul 34752, Türkiye; 3 Department of Health Care Management, Faculty of Health Sciences, Mus Alparslan University, Mus 49250, Türkiye; b.yetim@alparslan.edu.tr

**Keywords:** HIV, sexually transmitted infections, stigma, sexual norms, religiosity

## Abstract

**Background/Objectives**: Whether country-level sexual norms, religious salience and belief, and HIV-related social distancing are independently associated with HIV and non-HIV STI burden beyond economic conditions remains uncertain. **Methods**: We analyzed harmonized cross-national data from 68 countries (WVS waves 1996–2021; outcomes matched through 2022) using hierarchical OLS with HC3 robust standard errors, PCA-derived normative components, and GDP-adjusted inequality (Net GINI). **Results**: Higher GDP was associated with lower burden (HIV: b = −1.063; STI: b = −0.537; both *p* < 0.001), whereas higher inequality was independently associated with HIV (b = 0.074, *p* = 0.014) but not STI burden (*p* = 0.381). The religious salience-and-belief component showed no independent association after economic factors and HIV-related social distancing were included (HIV: *p* = 0.791; STI: *p* = 0.101). Sexual-attitude permissiveness was associated with HIV before social distancing entered the model (b = 0.461, *p* = 0.014) but not afterward (*p* = 0.922). Greater social distancing was inversely associated with measured burden (HIV: b = −3.157, *p* = 0.002; STI: b = −1.561, *p* < 0.001), but these associations attenuated to non-significance after adjustment for World Bank region (HIV: *p* = 0.745; STI: *p* = 0.187). **Conclusions**: Lower social distancing may facilitate testing and disclosure, increasing epidemiological visibility rather than true transmission. Programs should integrate stigma reduction into confidential testing and clinician training, anticipating short-term rises in recorded cases where testing improves.

## 1. Introduction

Worldwide, the number of people living with HIV has reached 40.8 million as of 2024, while more than 1 million new cases of four major sexually transmitted infections (STIs), namely, syphilis, gonorrhea, chlamydia, and trichomoniasis, have been reported per day. The components of annual mortality burden are approximately 630,000, 1.3 million, 350,000, and 230,000 for HIV (2024), viral hepatitis (2022), cervical cancer (2022), and syphilis (2022), respectively [1,2]. This burden reflects not only biomedical outcomes but also socioeconomic impacts exacerbated by stigmatization, discrimination, and psychological distress [3,4].

Although absolute income level (GDP per capita) influences testing, treatment, and prevention capacity, income inequality can particularly increase HIV burden through the exclusion of marginal groups, low social trust, and institutional fragility [5]. In environments faced with rapidly increasing inequality, this exclusion manifests through intermediary channels such as the proliferation of sex work [6], shared needle drug use and lack of access to harm-reduction services (e.g., needle–syringe programs, OST) [7], housing insecurity and overcrowded living spaces [8], male-dominated temporary work clusters in mining/port/highway corridors through irregular migration [9], and service disconnection due to police raids and short-term incarceration cycles [10]. These conditions intensify contact networks, weaken protective behaviors, and delay early diagnosis. Therefore, distinguishing the unique effects of absolute income and income distribution is essential: while high GDP per capita enhances the capacity of health systems, the level of inequality is a determinant of service access and social cohesion [11]. Conversely, the systematic effect of social norms and stigmatization, which are distinct from socioeconomic risk mechanisms, on testing behaviors constitutes another analytical dimension. Rigid sexual norms and stigmatization lead to avoidance of testing and concealment of status [12]. Thus, reducing stigmatization may lead to a short-term increase in measured prevalence due to the resultant increases in testing coverage and safe disclosure. This pattern may partly reflect an ascertainment effect [13]. Despite the influence of these structural and systemic factors, a number of studies [14,15] tend to associate changes in HIV prevalence with those in individual behavioral patterns. While interpretations explain changes in casual or premarital sexual behaviors through the loosening of norms [16], evidence indicates that the effect of these behaviors is interconnected with stigmatization, testing coverage, and safe access to services [17]. However, narratives of moral loosening alone are insufficient for explaining any epidemiological pattern [18]. This framework establishes the necessity outlined in the current study to objectively examine the relationships of social sexual norms, religiosity, and HIV-related stigmatization with HIV and non-HIV STI burdens while controlling for economic development and inequality.

The primary objective of this study is to examine whether country-level differences in HIV and non-HIV STI burden are associated with sexual-attitude permissiveness, religious salience and belief, and HIV-related social distancing after accounting for economic development and income inequality. Using harmonized data from 68 countries, we also examine whether coefficient attenuation after adding social distancing is compatible with an exploratory indirect-association pattern. The study contributes by evaluating structural, normative, and surveillance-related factors within a single cross-national ecological framework while distinguishing measured burden from potential differences in epidemiological visibility.

## 2. Materials and Methods

This study is ecological and cross-sectional in nature and conducted at the country level. Each country is represented by its most recent wave of data from the World Values Survey (WVS). The source WVS file contained 443,488 distinct respondents [19,20]. We excluded 29 records in which all 11 selected variables were coded with negative WVS missing-value codes, leaving 443,459 respondents. No duplicate respondent identifiers remained. Across the selected variables, these respondents contributed 3,070,950 valid item-level responses before country–year aggregation, and were linked to health and economic indicators by country and year. By design, this approach limits causal inference, does not capture temporal dynamics, and underrepresents marginalized populations. Nevertheless, it is suitable for examining macro-level associations between cultural factors and health outcomes. Country-level associations were not interpreted as estimates of individual-level behavior; proposed mechanisms involving testing, disclosure, or care-seeking are treated as hypotheses rather than as behaviors directly observed in this study.

### 2.1. Study Variables and Data Preprocessing

The first dependent variable is HIV prevalence among individuals aged 15–49 years per 100,000 population, reported as GBD 2021 estimates from IHME. High values indicate a high proportion of people living with HIV in society, while low values indicate that the epidemic has remained relatively contained. Second, STI incidence was obtained from data from the Global Burden of Disease conducted by the Institute for Health Metrics and Evaluation (IHME) [21] and encompasses STIs apart from HIV (e.g., chlamydia, gonorrhea, and syphilis). Data are reported as the age-standardized incidence rate of sexually transmitted infections excluding HIV, per 100,000 population. High STI values indicate worsening infectious disease burden, weak protective behaviors, and additional strain on health systems. GDP per capita, used at the macro level [22], represents the economic welfare levels of countries, while the GINI coefficient [23,24] reflects inequality in income distribution. High GINI values indicate intense inequality, while low values point to equitable distribution. Net GINI was defined as the residual from regressing GINI on log GDP per capita. This residualization was used to express inequality net of economic development for interpretability; by the Frisch–Waugh–Lovell theorem, when log GDP is included in the same model, the coefficient for residualized GINI is numerically identical to the partial coefficient obtained by entering raw GINI and log GDP simultaneously. GINI was retained because it provided broad comparable cross-national coverage for the study sample. For this reason net GINI and log GDP were included together in the first model. Additionally, data on condom use during high-risk sexual intercourse, an independent variable, as reported by the Joint United Nations Program on HIV/AIDS [25], were included as a fundamental behavioral indicator for the prevention of HIV and STI transmission; however, this indicator was excluded from analyses, because it appeared in only 3.6% of country–year observations; Supplementary Table S3 lists the variables excluded for critical missingness. Table 1 presents the variables used in the research models. Descriptive statistics for the normative and attitudinal items prior to missing-data handling are reported in Supplementary Table S1, and their distribution across country–years is shown in Supplementary Figure S1.

The independent variables used in the research consist of cross-sectional format data obtained from surveys conducted in specific years in countries and are presented with WVS codes. The normative variables derived from these surveys reflect attitudes toward sexuality and religious values. Among sexuality-related variables, F118 (Homosexuality), F119 (Sex Work), F132 (Casual Sex), and F135A (Premarital Sex) are coded using a 10-point scale (1–10), in which high values indicate that the behaviors under study are viewed as acceptable in society, while low values indicate conservative norms. For variables A006 (Importance of Religion) and D031 (Importance of Religious Belief in Marriage), which were reverse-coded, high scores indicate that religion plays a strong role in life, while low scores reflect secular tendencies. The variable A124_07 (Neighbors with AIDS) is a single general-population social-distance (“would not want as neighbors”) item; high values indicate greater social distancing toward people living with HIV/AIDS (PLHIV) and low values indicate greater acceptance. We therefore treat this item throughout as a measure of general-population social-distance attitudes toward PLHIV rather than as a comprehensive index of HIV-related stigma. For the variable F050 (Belief in God), high values indicate widespread belief in God, while low values demonstrate increased rates of non-belief. Because A006 and F050 assess the subjective importance of religion and belief in God, respectively, their PCA-derived component is interpreted as a measure of religious salience and belief rather than as a comprehensive index of multidimensional religiosity. D003 (Religious Sharing with Spouse) and D031 (Importance of Religious Belief in Marriage) were evaluated during preprocessing but excluded from all analytical models because of critical missingness. The scales used for the variables are as follows:

- **A006 (Importance of Religion):** 1 = *Very important*, 2 = *Rather important*, 3 = *Not very important*, 4 = *Not at all important* (this is original coding, but was reverse-coded as 5−i, where high scores indicate greater importance).
- **A124_07 (Neighbors With AIDS):** 1 = *Would not like as neighbor*, 0 = *Not mentioned*.
- **F118, F119, F132, and F135A (Sexual Norms):** 1 = *Never justifiable*, 10 = *Always justifiable*.
- **F050 (Belief in God):** 1 = *Yes*, 0 = *No*.
- **D003 (Religious Sharing With Spouse):** 1 = *Shares*, 0 = *Does not share* (preprocessing only; excluded from analytical models).
- **D031 (Importance of Religious Belief in Marriage):** 1 = *Very important*, 2 = *Important*, 3 = *Not very important*, 4 = *Not at all important* (this is original coding but was reverse-coded as 5−i, where high scores indicate greater importance) (preprocessing only; excluded from analytical models).

Negative codes commonly used across items in the WVS dataset were cleaned before analysis. These codes are −5 = *Missing/Not available*, −4 = *Not asked in survey*, −3 = *Not applicable*, −2 = *No answer*, and −1 = *Don’t know*. Only valid (positive) values were included in the analysis.

The dataset for analytical models was primarily prepared through country–year averages derived from WVS respondent-level data summarized per country and year and converted to panel format. Sample weighting provided by the WVS was not used when creating the panel data. This was an analytical choice intended to maintain a uniform aggregation procedure across countries and variables; however, unweighted means may be sensitive to within-country sample composition, and the absence of a weighted sensitivity analysis is acknowledged as a limitation.

In the data integration process, data prior to 1990 were excluded due to limited reliable measurements of dependent variables during this period. IHME provides standardized, globally comparable estimates of HIV prevalence and STI incidence beginning in 1990; however, consistent and methodologically reliable data are not available for earlier years. To create a cross-sectional dataset from the panel structure, the most recent year data for each country was selected. This approach initially provided a sample of 108 countries. However, missing rates for Importance of Religion, HIV-related social distancing, Acceptance of Homosexuality, Acceptance of Sex Work, Belief in God, HIV Prevalence, STI Incidence, and GDP Per Capita ranged from 0.9% to 9.3%, while high missing rates were detected in sexuality-themed questions (Acceptance of Casual sex: 42.6%, Acceptance of Premarital Sex: 27.8%) and the GINI coefficient (41.7%).

To minimize sample loss, the study attempted to use the last observation carried backward (LOCB) approach. LOCB implementation decreased the sample to 88 countries, but temporal transfer analysis revealed critical problems. Requirements for backward data transfer extending up to 21 years for GDP per capita in Venezuela, 24 years for HIV/STI indicators in the Czech Republic, and 17 years for social attitude variables in Egypt were identified. Thus, the study deemed that 20 and 14 countries required a temporal transfer of more than 5 years for at least one variable and more than 10 years, respectively. This long-term backward carrying would compromise temporal consistency and create a systematic downward bias, especially for rapidly liberalizing sexual attitudes and volatile economic indicators (Supplementary Table S2; see also Supplementary Material, Missing Value Analysis). Therefore, LOCB implementation was abandoned. Instead, a cross-sectional dataset of 68 countries was created by excluding 20 countries with missing data in the 10 core variables that exhibit low missingness (i.e., Importance of Religion, Happiness, Health Status, HIV-related social distancing, Acceptance of Homosexuality, Acceptance of Sex Work, Belief in God, HIV, STI, GDP Per Capita). Variables obtained from non-WVS sources (i.e., HIV prevalence, STI incidence, GDP per capita, and the GINI coefficient) were integrated with the same country–year matches. Across the final 68 countries, the WVS waves used were predominantly recent: 46 countries (68%) drew on waves conducted in 2015 or later (median wave year 2018; range 1996–2021). The wave year for each country is reported in Supplementary Table S6, and the distribution by region in Supplementary Table S5.

After integration, missing data for 23 countries (33.8%) were detected in the GINI coefficient initially obtained from World Bank indicators. To address these gaps, alternative reliable sources were examined, and GINI data were obtained for the 23 countries (Table S4, Supplementary Material). These data were compiled from the World Bank’s Poverty and Inequality Platform (nearest year data if current data were unavailable), OECD Income Distribution Database, International Monetary Fund, and national statistical institutes. During the data compilation process, the study attempted to adopt the World Bank’s methodological approach [26], with preference for income- and consumption-based GINI values for developed and developing countries, respectively. However, this standardization could not be achieved due to constraints in data accessibility. The reason is that the heterogeneity of data sources (combination of income- and consumption-based measurements) and temporal mismatches (use of data from 2 to 8 years earlier for certain countries) introduced methodological limitations. Nevertheless, this approach enabled the inclusion of data on economic inequality indicators for the 68 countries included in the analysis. The Supplementary Material provides detailed information on the completion process of GINI data from alternative sources.

The 68 countries span a wide geographic and cultural range, from Eastern Europe to Latin America and from Sub-Saharan Africa to Southeast Asia; their distribution by World Bank region, together with region-specific disease burdens, is reported in Supplementary Table S5, and country-level values are listed in Supplementary Table S6. This distribution reflects World Values Survey coverage rather than the global distribution of disease burden: Sub-Saharan Africa, where most of the world’s HIV burden is concentrated, contributes only 9 of the 68 countries, so the HIV findings are weighted toward Europe and Central Asia, Latin America, and East and Southeast Asia and may not generalize to the highest-burden settings. We did not include regional dummy variables in the primary models because, with seven regions and several sparsely populated categories (e.g., only two countries in North America), six dummies would have consumed substantial degrees of freedom and compromised parsimony at n = 68, while economic development and inequality already capture much interregional and intraregional heterogeneity [27,28]. Because region is nonetheless a dominant axis of HIV variation, we additionally estimated a World Bank region fixed-effect specification as a sensitivity analysis (see Section 3; Supplementary Table S7).

### 2.2. Data Analysis

Python 3.11.15 was used for analysis, with the pandas, numpy, statsmodels, scipy, and matplotlib libraries utilized for dataset preparation and analyses [29,30,31,32]. Logarithmic transformation was applied to HIV prevalence, STI incidence, and GDP per capita due to right-skewed distributions (skewness > 1.7). Normality assumption was evaluated using the Shapiro–Wilk test, and distributions were observed to approach normality after log transformation. The Net GINI (GDP-adjusted) variable was created using residuals obtained from regressing GINI on log(GDP). The distribution of residuals and heteroscedasticity were examined using the Anderson–Darling and Breusch–Pagan tests, respectively. The possibility of multicollinearity was evaluated through variance inflation factor (VIF) analysis, and variable combinations with VIF values > 10 were identified. Principal component analysis (PCA) was performed to reduce this problem. In testing the main hypothesis, hierarchical regression models were established, while heteroskedasticity-consistent (HC3) robust standard errors [33] were used to provide robust inference in the presence of potential heteroskedasticity in the modest country-level sample. Spearman’s and Pearson’s correlations were used in correlation analyses. Statistical significance was evaluated at the 5% level.

### 2.3. Hierarchical Model Specification

Hierarchical regression was structured in theoretical blocks:

- **Block 1 (economic structure):** balances structural determinants by including the logarithm of GDP per capita and GDP-adjusted GINI;
- **Block 2 (normative–cultural factors):** includes the religiosity and sexual-attitude permissiveness components, which represent the societal value system in the same step. This block was selected due to the distinct negative correlation between components and their shared conceptual framework as normative and cultural factors; and
- **Block 3 (HIV-related social distancing):** includes HIV-related social distancing and examines whether associations with normative variables persist after its inclusion and whether coefficient attenuation is compatible with an exploratory indirect-association pattern.

Each block’s contribution was reported with R^2^, ΔR^2^, adjusted R^2^, and Wald’s χ^2^ values; Akaike’s Information Criterion (AIC) and Bayesian Information Criterion (BIC) were additionally provided for model comparability.

### 2.4. Model Selection and Rationale for Excluding Alternative Approaches

Model method selection was based on two constraints: the coefficient-attenuation pattern involving HIV-related social distancing and sample size (n = 68). Given that cross-sectional data could not meet temporal precedence and sequential ignorability assumptions, and latent measurement models require numerous free parameters, formal mediation/SEM was not preferable as the primary analysis. Instead, HIV-related social distancing was added in Block 3, while interpretation was conducted through sensitivity patterns. Given the small sample, the study excluded high-degree polynomials, splines/GAM, and intensive interactions, because they increased overfitting risk and limited improvement relative to the BIC penalty. Multilevel/panel and instrumental variable approaches were also deemed inapplicable due to lack of repeated observations across time and lack of strong exogenous instruments. Therefore, the study adopted a three-block linear hierarchical model as the main analysis following the principles of parsimony and interpretability. The three-block hierarchical models for HIV and STI constituted the primary analytical specification. PCA construction, functional-form comparisons, GINI residualization, and exploratory indirect-effect decomposition are reported as secondary and sensitivity analyses, together with additional robustness checks that add a wave-year control, restrict the sample to 2015-or-later waves, add World Bank region fixed effects, and adjust the social-distancing–HIV association for antiretroviral therapy (ART) coverage as a proxy for the completeness of the HIV detection-and-treatment cascade (Supplementary Tables S7 and S8).

### 2.5. Exploratory Indirect-Effect Analysis

This hypothesis was separately tested, because the attenuation of sexual-attitude permissiveness coefficients with the addition of HIV-related social distancing in hierarchical models produced a coefficient-attenuation pattern motivating an exploratory indirect-effect decomposition. Toward this end, an exploratory indirect-effect decomposition (product-of-coefficients) with GDP per capita and GINI as covariates was applied following the Baron and Kenny [34] approach. The Sobel test was used to evaluate the significance of indirect effects, and the findings were further strengthened by calculating bias-corrected and accelerated (BCa) 95% confidence intervals through 5000-iteration nonparametric bootstrap to overcome distributional assumptions. Because the data were cross-sectional and ecological, temporal ordering and sequential ignorability could not be established, and the indirect-effect analysis was therefore interpreted descriptively and not as evidence of causal mediation.

## 3. Results

Figure 1 presents country-level means for the final analytical cross-section of 68 countries, using each country’s selected WVS wave. Item-specific respondent counts are reported below.

On the original A006 coding (1 = Very important, 4 = Not at all important), the mean Importance of Religion score was 1.93 ± 0.66 (n = 98,266). The country-level proportion of respondents indicating that they would not want people with AIDS as neighbors, based on a dichotomously coded WVS item, averaged 0.41 ± 0.25 (n = 98,194), indicating substantial variation in HIV-related social distancing across countries. Examination of normative indicators indicated that Acceptance of Homosexuality, Acceptance of Sex Work, and Acceptance of Casual Sex reached 3.39 ± 1.96 (n = 95,816), 2.71 ± 1.09 (n = 96,868), and 3.37 ± 1.46 (n = 67,289), respectively, which are low levels. In contrast, Acceptance of Premarital Sex exhibited a higher average (4.75 ± 2.10; n = 84,088), thus revealing that this behavior is more accepted compared with other sexual norms. Belief in God reached 0.85 ± 0.19 (n = 96,581), which implies that the majority (85%) believed in God. Among the dependent variables, STI incidence (age-standardized rate) and HIV prevalence were 4.49 ± 6.76 and 1586 ± 4972 (per 100,000), respectively. Economic indicators demonstrated a mean per capita GDP of 12,727 USD ± 16,582, and a GINI coefficient of 38.6 ± 9.4 (n = 68), which indicate substantial income inequalities among countries (Figure 1).

Importance of Religion (A006) was reverse-coded using 5 − score so that higher values indicated greater religious importance. D031 underwent the same preprocessing when available but was excluded from all analytical models because of critical missingness. This directional harmonization was applied before constructing the religiosity component from A006 and F050. Following these transformations, distribution and correlation analyses were conducted.

Distribution analyses obtained from normative and structural indicators pointed to the necessity of logarithmic transformation for HIV (skewness = 4.17), STI (2.20), and Per Capita GDP (1.84). After applying log transformations, distributions became notably more symmetric [log(HIV) = 0.66; log(STI) = 0.86; log(GDP) = 0.02]. Shapiro–Wilk normality tests indicated that log(GDP) met the normal distribution assumption (W = 0.976, *p* = 0.204), but deviations from normal distribution were still observed for the crucial dependent variables (log(HIV): W = 0.945, *p* = 0.005; log(STI): W = 0.893, *p* < 0.001).

Following distributional assessments, correlation analyses were performed using appropriate methods based on each variable’s distribution. Pearson’s correlations were calculated for normally distributed and log-transformed variables, while Spearman’s correlations were used for variables with nonnormal distributions. This methodological approach ensured a valid examination of relationships among variables and provided preliminary insights for multicollinearity assessment prior to hierarchical regression modeling. Figure 2 presents the full correlation matrix.

Correlation analysis revealed distinct patterns among the variables. A strong positive correlation was observed between HIV prevalence and STI burden (ρ = 0.656, *p* < 0.001), whereas both health indicators exhibited negative associations with Per Capita GDP (STI: ρ = −0.562, *p* < 0.001). This result confirms that economic development is associated with low disease burden. Religion-related variables demonstrated strong internal consistency, with Importance of Religion and Belief in God displaying high correlations (*r* = 0.813, *p* < 0.001). These religiosity indicators exhibited consistent negative relationships with measures of sexual-attitude permissiveness, particularly Acceptance of Premarital Sex (Importance of Religion: ρ = −0.799, *p* < 0.001; Belief in God: ρ = −0.718, *p* < 0.001) and Acceptance of Homosexuality (Importance of Religion: *r* = −0.634, *p* < 0.001; Belief in God: *r* = −0.564, *p* < 0.001). Furthermore, both religiosity indicators displayed negative correlations with Per Capita GDP (Importance of Religion: ρ = −0.534, *p* < 0.001; Belief in God: ρ = −0.533, *p* < 0.001). Indicators of sexual-attitude permissiveness displayed strong positive intercorrelations; the highest associations were found between Acceptance of Sex Work and Acceptance of Casual Sex (ρ = 0.936, *p* < 0.001) and between Acceptance of Homosexuality and other measures of sexual-attitude permissiveness (Acceptance of Sex Work: *r* = 0.853, *p* < 0.001; Acceptance of Casual Sex: ρ = 0.843, *p* < 0.001; Acceptance of Premarital Sex: ρ = 0.823, *p* < 0.001). These variables demonstrated positive relationships with economic development, particularly with GDP per capita: both Acceptance of Premarital Sex (ρ = 0.663, *p* < 0.001) and Acceptance of Homosexuality (ρ = 0.639, *p* < 0.001) (Figure 2).

The absence of a significant correlation between GDP Per Capita and the GINI coefficient (ρ = −0.103, *p* > 0.05) indicates that they represent different dimensions. However, to express income inequality net of economic development, Net GINI (GDP-adjusted) was introduced. This transformation was accomplished by regressing the GINI coefficient on log(GDP per capita) and using residuals as an indicator of relative inequality:

$$\begin{array}{cc} \mathrm{Net}\;{\mathrm{GINI}}_{i}\;=\;{\mathrm{GINI}}_{i}\; & -\;(\alpha \;+\;\beta \;\times \;\log\;\mathrm{GDP}\;\mathrm{per}\;{\mathrm{capita}}_{i})\;\\ = & \;{\mathrm{GINI}}_{i}\;-\;(48.00\;+\;1.08\;\times \;\log\;\mathrm{GDP}\;\mathrm{per}\;{\mathrm{capita}}_{i}) \end{array}$$

This approach measures each country’s deviation from the expected inequality after considering its level of economic development. Positive residuals indicate high inequality compared with countries with similar GDP levels, whereas negative residuals indicate relatively low inequality. In the current sample, GDP explained only 2.2% of variance in GINI (R^2^ = 0.022), indicating that inequality is mainly independent of economic development. The highest positive residuals are observed for Nicaragua (+27.49), Saudi Arabia (+25.76), and South Africa (+24.23), whereas the lowest are noted for Ukraine (−13.51), Slovenia (−12.15), and Belarus (−11.30). Residualizing GINI on log(GDP) eliminated the weak correlation between GDP and GINI, enabling simultaneous interpretation of the independent associations of both variables. By the Frisch–Waugh–Lovell theorem, entering log(GDP) together with this GINI residual yields a partial coefficient for inequality that is numerically identical to the one obtained by entering log(GDP) and the raw GINI jointly; the residualization is therefore an interpretive device that expresses inequality net of economic development rather than an additional statistical adjustment. Analysis of inequality dimensions independent of economic development became possible because this procedure preserved 97.88% of GINI variation, thus enabling testing for the independent association of income inequality with HIV prevalence and STI burden.

The strong relationships observed in correlation analysis led to the anticipation of high levels of multicollinearity among variables. Therefore, multicollinearity analysis based on a model constructed using all variables for inclusion in hierarchical regression revealed a serious problem, as anticipated. High VIF values among the variables of religion (Importance of Religion and Belief in God: VIF > 70) and sexual norm (Acceptance of Homosexuality and Acceptance of Sex Work: VIF > 29), as well as log(GDP) (VIF = 68) indicated that these indicators should not be entered separately in the same multivariable model. High correlations also confirmed this problem (Importance of Religion/Belief in God: *r* = 0.813; Acceptance of Homosexuality/Acceptance of Sex Work: *r* = 0.853; GDP/Homosexuality: *r* = 0.722). PCA was therefore used to summarize their shared variance and reduce multicollinearity. The sexual-attitude permissiveness component was derived from Acceptance of Homosexuality and Acceptance of Sex Work (92.7% of variance explained), while the religiosity component was constructed using Importance of Religion and Belief in God (90.7% of variance explained). After this transformation, VIF values decreased to acceptable levels, thus solving the problem (Per Capita GDP: 5.71, HIV-related social distancing: 6.34, sexual-attitude permissiveness component: 2.97, religiosity component: 2.01, and GDP-adjusted GINI: 1.18). Additionally, the low VIF value of GDP-adjusted GINI indicated that it is orthogonal to other predictors and can provide an independent contribution to the model (Figure 3).

Through correlation analysis conducted with logarithmically transformed or component-constructed model variables for hierarchical regression, intervariable relationships became increasingly evident. While the strong positive correlation between HIV prevalence and STI burden (*r* = 0.709, *p* < 0.001) was preserved, both health indicators exhibited negative relationships with per capita GDP (HIV: *r* = −0.551, *p* < 0.001; STI: *r* = −0.620, *p* < 0.001), a pattern consistent with lower measured disease burden at higher GDP levels. The religiosity component (PC1) is positively correlated with HIV prevalence (*r* = 0.431, *p* < 0.001) and STI burden (*r* = 0.300, *p* = 0.013) but strongly, negatively correlated with sexual-attitude permissiveness (*r* = −0.574, *p* < 0.001). Additionally, the religiosity component is negatively correlated with per capita GDP (*r* = −0.583, *p* < 0.001) but positively correlated with GDP-adjusted GINI (*r* = 0.280, *p* = 0.021). The sexual-attitude permissiveness component is positively correlated with per capita GDP (*r* = 0.659, *p* < 0.001) but negatively correlated with HIV-related social distancing (*r* = −0.509, *p* < 0.001) and STI burden (*r* = −0.274, *p* = 0.024). The positive relationship of HIV prevalence with GDP-adjusted GINI (*r* = 0.366, *p* = 0.002) and negative relationship with HIV-related social distancing (*r* = −0.306, *p* = 0.011) revealed that disease burden is associated not only with economic factors but also with social inequality and social-distancing dynamics.

Linear, polynomial (quadratic), and cubic models for HIV and STI outcomes were systematically compared using the same predictor set. Model performance was evaluated according to multiple criteria, including the coefficient of determination (R^2^), adjusted R^2^, Akaike’s information criterion (AIC), Bayesian information criterion (BIC), and visual inspection of fitted curves. Given the limited sample size (n = 68), particular attention was paid to maintaining parsimony and avoiding overfitting.

Linear models achieved moderate explanatory power (HIV: *R*^2^ = 55.4%; STI: *R*^2^ = 53.1%) with parsimonious parameter structures (six parameters each). Polynomial models displayed modest improvements (HIV: *R*^2^ = 63.2%, STI: R^2^ = 63.4%) but required 11 parameters. Cubic models demonstrated very high *R*^2^ values (HIV: R^2^ = 94.1%, STI: R^2^ = 94.8%) but at the cost of excessive complexity (56 parameters). BIC comparison strongly favored linear models: HIV: linear: 269.09 vs. polynomial: 287.34 vs. cubic: 341.67 and STI: linear: 155.85 vs. polynomial: 167.12 vs. cubic: 219.89. The dramatic increase in BIC for cubic models (HIV: +72.58 points, STI: +64.04 points) indicates severe overfitting penalties that outweigh goodness-of-fit gains (Figure 4).

Visual inspection through locally estimated scatterplot smoothing (LOESS) overlays revealed that despite the existence of a number of nonlinear patterns, particularly in GDP–outcome relationships, these deviations were not sufficiently systematic to justify the cost of complexity. LOESS curves generally tracked close to linear trends, with confidence intervals encompassing the linear fits for the majority of the data range. The BIC-based model selection process clearly supports linear specification. With only 68 observations, cubic models that require 56 parameters represent a data-to-parameter ratio of approximately 1.2:1, which is methodologically inappropriate and may produce unstable, nongeneralizable estimates. Polynomial models, while offering a degree of improvement in fit, do not provide sufficient gain to justify the increased complexity when parsimony is considered. Thus, linear hierarchical regression models provide the optimal balance between explanatory power and parsimony for this cross-national ecological analysis. The slight non-linearities observed do not warrant the substantial increases in complexity required by higher-order polynomial specifications (Figure 4).

The hierarchical regression model was structured in sequential theoretical blocks to evaluate associations of socioeconomic and cultural factors with disease burden. Model 1 (Block 1: economic structure) includes log(GDP per capita) and GDP-adjusted GINI as control variables, establishing the economic baseline before sociocultural variables are added. Model 2 (Block 2: normative–cultural factors) adds the religiosity component (Religiosity PC1), which captures religious salience and belief, together with the Sexual-Attitude Permissiveness PC1. Because these components represent related but distinct aspects of the societal value–norm structure (r = −0.574), they were entered in the same step. Model 3 (Block 3: HIV-related social distancing) examines whether associations with normative variables persist after social distancing is included and whether coefficient attenuation is compatible with an exploratory indirect-association pattern (Table 2).

**Economic block (Model 1):** This block represents the control variables. Per capita income (log(GDP)) demonstrates strong and consistent negative associations with both dependent variables (HIV: b = −0.946 → −1.063, 95% CI [−1.473, −0.654] in Model 3; STI: b = −0.452 → −0.537, 95% CI [−0.722, −0.351] in Model 3; *p* < 0.001) in all models. This result represents a strong elasticity finding, illustrating that a 10% increase in GDP is associated with approximately 9.6% and 5.0% decreases in HIV and STI, respectively. Additionally, GDP-adjusted GINI is positive and significant for HIV (b ≈ 0.074–0.087; Model 3 95% CI [0.015, 0.132]; *p* = 0.014–0.005) but non-significant for STI. Simply put, even after controlling for GDP, HIV rates are higher in places with high income inequality, but the same does not apply to other STIs. Economic structure alone accounts for a substantial portion of explained variance (HIV: *R*^2^ = 0.438; STI: *R*^2^ = 0.396; Table 2).

**Normative–cultural block (Model 2)**: The religiosity component (Religiosity PC1), which captures religious salience and belief, showed no independent association with HIV or STI burden (HIV: b = 0.187, 95% CI [−0.268, 0.641], *p* = 0.421; STI: *p* = 0.579). In contrast, the sexual-attitude permissiveness component (Sexual-Attitude Permissiveness PC1) is positive and significant for HIV (b = 0.461, 95% CI [0.094, 0.828], *p* = 0.014) but not for STI. This step provides small-to-moderate additional explanatory power in the HIV model (Δ*R*^2^ = 0.041; Wald’s χ^2^ = 7.08; *p* = 0.029) but does not provide a notable improvement in STI (Δ*R*^2^ = 0.033; *p* = 0.247).

**Social-distancing block (Model 3):** When HIV-related social distancing is added, two important patterns emerge:

- HIV-related social distancing displays a strong and negative relationship (HIV: b = −3.157, 95% CI [−5.181, −1.133], *p* = 0.002; STI: b = −1.561, 95% CI [−2.399, −0.723], *p* < 0.001). A 10-point increase in HIV-related social distancing at the country level (e.g., from 30% to 40%) is seemingly associated with approximately 27% and 14–15% lower burdens for HIV and STI, respectively. This block provides the largest increase in explanatory power in both models (HIV: Δ*R*^2^ = 0.075; STI: Δ*R*^2^ = 0.102) and significantly improves model fit (HIV: Wald’s χ^2^ = 9.34; *p* = 0.002; STI: Wald’s χ^2^ = 13.32; *p* < 0.001). Decreases in AIC/BIC also confirmed the net contribution to the model (HIV AIC: 264.3 → 255.8; STI AIC: 153.9 → 142.5).
- The association of sexual-attitude permissiveness is attenuated and changes direction. When HIV-related social distancing is added, the positive association of the sexual-attitude permissiveness component (Sexual-Attitude Permissiveness PC1) with HIV is attenuated to the null (b = −0.025, 95% CI [−0.515, 0.466], *p* = 0.922). It similarly remains nonsignificant for STI (b = −0.107, 95% CI [−0.315, 0.101], *p* = 0.314). This finding implies that the association between sexual-attitude permissiveness and disease burden is accompanied by HIV-related social distancing as a shared-variance (suppressor) channel; when shared variance between permissive norms and lower social distancing is controlled, the independent association of sexual-attitude permissiveness disappears.

The attenuation of the sexual-attitude permissiveness coefficient after the inclusion of HIV-related social distancing motivated an exploratory indirect-effect decomposition. Although this was not a direct research question, based on this finding, the coefficient of sexual-attitude permissiveness in the HIV model was *b* = 0.461 (*p* = 0.014) in Model 2 but decreased to *b* = −0.025 (*p* = 0.922) in Model 3 after the addition of HIV-related social distancing. Similarly, in the STI model, it declined from *b* = 0.133 (*p* = 0.160) to *b* = −0.107 (*p* = 0.314). These patterns showed that the sexual-attitude permissiveness coefficient attenuated after HIV-related social distancing was added. To describe this attenuation, an exploratory indirect-effect decomposition (product-of-coefficients) was performed following the Baron and Kenny approach, controlling for GDP per capita and the GINI coefficient. This decomposition was interpreted descriptively rather than as evidence of causal mediation and was accompanied by bootstrap confidence intervals.

The decomposition is consistent with an indirect association in which HIV-related social distancing accompanies the ecological relationship between sexual-attitude permissiveness and measured disease burden. Because the design is cross-sectional and ecological, we interpret this decomposition descriptively rather than as a formal causal test of mediation. While the total effect of sexual-attitude permissiveness was positive and significant for the HIV model (*b* = 0.393, *p* = 0.006), the direct effect decreased nearly to zero (*b* = 0.007, *p* = 0.974) and lost statistical significance after HIV-related social distancing was included. The indirect effect (θ = 0.386) was found significant (Sobel test; z = 2.296, *p* = 0.022). An increase of 0.386 on the log-scale corresponds to an increase of approximately 47% (given that exp(0.386) ≈ 1.47 → 47%). The descriptive indirect/total ratio is reported in Supplementary Table S9 for HIV only; because the total association is modest and the design is cross-sectional and ecological, this ratio is unstable and should not be interpreted causally. The STI model exhibited a similar pattern. Although the total effect was not significant at the 5% level (*b* = 0.155, *p* = 0.083), the direct effect, once again, became nonsignificant after HIV-related social distancing was included (*b* = −0.003, *p* = 0.974). The indirect effect was significant (Sobel z = 2.231, *p* = 0.026); however, because the STI total effect is weak and not significant at the 5% level, the descriptive indirect/total ratio is unstable and is not reported (a point estimate above 100% would reflect numerical instability rather than a substantive suppression effect). In the exploratory indirect-association pattern, sexual-attitude permissiveness is associated with lower HIV-related social distancing (*b* = −0.126, *p* < 0.001), and lower HIV-related social distancing is in turn associated with higher measured disease burden (HIV: *b* = −3.063, *p* = 0.003; STI: *b* = −1.255, *p* = 0.004). A 10-point increase in HIV-related social distancing is associated with reductions of approximately 26–27% and 12% in HIV prevalence and STI burden, respectively. Overall, the product-of-coefficients estimate is compatible with an indirect association between sexual-attitude permissiveness and measured disease burden via HIV-related social distancing; this seemingly paradoxical pattern is compatible with higher testing and better case detection where social distancing is lower (Table 3).

To confirm the pattern and test the robustness of the findings, 5000-iteration nonparametric bootstrap was used to calculate BCa 95% confidence intervals. The results were similar to those of the Sobel test. For HIV, the indirect effect was significant (θ = 0.386; BCa 95% CI [0.071, 0.790]; *p*_boot_ = 0.001), whereas the direct effect became nonsignificant (c′ = 0.007; *p* = 0.974), indicating a pattern in which the direct effect was no longer significant after HIV-related social distancing was included. For STI, the indirect effect was also significant (θ = 0.158; BCa 95% CI [0.061, 0.307]; *p*_boot_ = 0.016). For STI, the descriptive indirect/total ratio is not reported: because the total effect is near zero, this ratio is numerically unstable and its bootstrap interval is uninformative, so we report only the indirect effect and its confidence interval. Exploratory indirect-effect analyses yielded results consistent with those of the hierarchical regression, in which sexual-attitude permissiveness coefficients attenuated after HIV-related social distancing was added.

Sensitivity analyses supported the robustness of the main associations (Supplementary Table S7). Adding a linear wave-year control left the HIV-related social-distancing coefficients essentially unchanged (HIV: *b* = −3.07, *p* = 0.001; STI: *b* = −1.58, *p* < 0.001), and the year term was non-significant (HIV: *p* = 0.20; STI: *p* = 0.70), indicating that secular trends did not drive the findings. Restricting the sample to countries with a 2015-or-later WVS wave (n = 46) preserved the social-distancing associations (HIV: *b* = −2.61, *p* = 0.024; STI: *b* = −1.85, *p* < 0.001). In a more demanding specification adding World Bank region fixed effects, the HIV-related social-distancing coefficients attenuated and lost significance (HIV: b = −0.364, 95% CI [−2.562, 1.833], *p* = 0.745; STI: b = −0.801, 95% CI [−1.989, 0.388], *p* = 0.187), while the GDP coefficient weakened but remained significant (HIV: b = −0.459, 95% CI [−0.889, −0.029], *p* = 0.037; STI: b = −0.406, 95% CI [−0.675, −0.138], *p* = 0.003). Because region is a principal axis of HIV variation and, at *n* = 68 with seven regions, region fixed effects are both demanding on degrees of freedom and strongly collinear with stigma, we interpret this attenuation as indicating that the social-distancing–burden association operates largely between rather than within regions—consistent with systematic between-region differences in both stigma climates and surveillance/detection infrastructure rather than as evidence against an ascertainment interpretation at the ecological level.

To probe whether differential case ascertainment might contribute to the inverse social-distancing–HIV association, we additionally adjusted for antiretroviral therapy (ART) coverage—the percentage of people living with HIV who are receiving treatment—as a proxy for the completeness of the HIV detection-and-treatment cascade and, in part, for differential survival (UNAIDS via the World Bank; available for 55 of the 68 countries). Higher HIV-related social distancing was associated with lower ART coverage (r = −0.30), and ART coverage was positively associated with measured HIV prevalence (b = 0.028, *p* = 0.003), patterns consistent with more complete case detection in higher-coverage settings. Adjusting for ART coverage attenuated the social-distancing coefficient by about 13% in the full matched sample (b = −3.67 to −3.18; both *p* < 0.001) and by roughly 34% when the sample was restricted to 2015-or-later waves (b = −2.48, *p* = 0.062, to b = −1.64, *p* = 0.12). The bootstrap indirect effect was negative but not statistically significant (full sample θ = −0.49, 95% CI [−1.38, 0.09]). Because ART coverage reflects both the detection-and-treatment cascade and survival, it may account for only a modest share of the association without fully explaining it, and a residual inverse association persists in the full sample (Supplementary Table S8).

## 4. Discussion

All associations reported in this section are ecological (country-level); any individual-level mechanisms are offered as interpretation and cannot be identified from aggregate data. Economic development was strongly correlated with lower HIV and non-HIV STI burdens across the 68 countries. In hierarchical models, a 10% increase in GDP per capita was associated with ≈9.6% lower HIV prevalence and ≈5% lower STI incidence (HIV: b for log(GDP) ≈ −1.06; STI: −0.54; *p* < 0.001), which underscores the centrality of system capacity and access to prevention, diagnostics, and treatment (Table 2). These patterns align with evidence that better-resourced health systems sustain broad testing and early treatment despite contextual differences in behavioral and network dynamics. Income inequality was independently associated with higher HIV prevalence over and above national income, whereas its association with STI incidence was null (HIV: b for net GINI ≈ 0.07–0.09, *p* ≤ 0.014; STI: not significant). This divergence is consistent with studies linking inequality to structurally mediated HIV risk—through precarious employment, housing insecurity, spatial segregation, and exclusion of key populations. Alternatively, many STIs circulate more diffusely and are detected earlier through routine sexual health services [35,36,37,38]. For policy, the inequality signal in HIV may serve as an early warning for settings with widening distributive gaps.

After accounting for economic development and HIV-related social distancing, the dimensions of religiosity captured here—the subjective importance of religion and belief in God—were not independently associated with HIV or non-HIV STI burden. Although simple correlations showed associations among religiosity, norms, and disease metrics, this salience-and-belief component did not explain additional variance in the multivariable models. This finding is consistent with literature emphasizing the importance of socioeconomic position, education, and gendered norms in cross-national HIV patterns, but should not be interpreted as excluding possible associations with other dimensions of religiosity [39,40,41]. Sexual-attitude permissiveness was positively associated with HIV in normative models but lost significance after the inclusion of HIV-related social distancing, while HIV-related social distancing was inversely associated with measured HIV and STI burdens and yielded the largest incremental gain in fit (HIV: ΔR^2^ ≈ 0.08; STI: 0.10; *p* ≤ 0.002; Table 2 and Table 3). Substantively, a 10-point increase in HIV-related social distancing corresponded to ≈27% lower measured HIV prevalence and ≈14–15% lower measured STI incidence—an ostensibly protective pattern that is compatible with an ascertainment effect. At the country level, lower average social-distancing scores co-occur with higher measured burdens—a pattern consistent with greater testing, safe disclosure, and earlier care-seeking, although these individual-level behaviors cannot be identified from aggregate data; conversely, higher social distancing co-occurs with lower measured burdens despite plausibly ongoing transmission [42,43,44,45]. Exploratory indirect-effect analyses were consistent with this interpretation: in the ecological data, the sexual-attitude permissiveness–HIV coefficient attenuated after HIV-related social distancing was included (indirect effect θ ≈ 0.39, Sobel *p* = 0.022; BCa 95% CI ≈ [0.07, 0.79]), with the direct effect becoming nonsignificant after HIV-related social distancing was included. This pattern is consistent with liberal climates co-occurring with lower social distancing and better detection rather than with higher true transmission, although the cross-sectional ecological design precludes causal confirmation. However, the role of HIV-related social distancing in HIV/STI surveillance remains understudied. While previous evidence supports similar ascertainment patterns [46,47], further research is required to fully elucidate the mechanisms through which social distancing influences surveillance outcomes versus actual disease transmission.

Taken together, the main findings redirect the explanatory emphasis from individual moral or behavioral narratives toward structural and measurement-related associations. Higher economic development was associated with lower measured HIV and STI burdens, while greater income inequality was associated with higher HIV burden net of income. The religious salience-and-belief component showed little independent association after structural and social-distancing factors were included, and HIV-related social distancing may influence who becomes visible in epidemiological data. Accordingly, short-term increases in detected cases after reductions in social distancing may reflect improved case finding rather than epidemiological deterioration, although this mechanism cannot be established from the present ecological data.

At the ecological level, more permissive sexual norms tend to co-occur with lower social distancing toward PLHIV and with higher measured burdens. A plausible—though, given the aggregate design, not directly testable—individual-level reading is that lower social distancing may facilitate routine testing, safe disclosure, and earlier care, so that measured burdens rise as a detection/ascertainment effect rather than through higher true prevalence. By the same logic, contexts with higher social distancing may foster test avoidance, concealment, and delayed care (e.g., through fear of job loss, social exclusion, or legal sanction), although these mechanisms operate at the individual level and cannot be confirmed here. Under such conditions, measured prevalence and incidence may appear lower when infections are less visible to health systems. The positive association between HIV and income inequality is potentially channeled through structural vulnerabilities (e.g., precarious employment, inadequate housing and insurance coverages, low health literacy, and spatial segregation). In contrast, per capita GDP indexes prevention, diagnostic, and treatment capacity, including laboratory infrastructure, free or low-cost testing, pre-exposure prophylaxis (PrEP) uptake, and access to antiretroviral therapy (ART). Taken together, the findings imply that normative climates may be more closely related to patterns of contact with the health system than to the underlying biology of transmission. Sign reversals and emergence of indirect-effect patterns when variables are entered blockwise (e.g., inequality, GDP, and norms) are consistent with these behavioral and measurement dynamics.

## 5. Conclusions

The findings indicate that HIV/STI burden is more closely associated with system capacity and safe access than with the sexual-attitude and religious-salience dimensions measured here after structural and social-distancing factors are considered. Lower HIV-related social distancing was associated with higher measured burdens, but this association was strongly attenuated after adjustment for World Bank region, indicating that it largely reflects between-region differences. Income inequality remained positively associated with HIV prevalence independent of per capita income. These results underscore that surveillance metrics may reflect not only underlying disease burden but also differences in access to testing, disclosure, and care. Therefore, reducing structural inequalities and HIV-related stigma remains important alongside biomedical prevention and treatment. Put simply, this study does not show that countries where religion is considered more important, or where belief in God is more widespread, have higher or lower levels of HIV or other sexually transmitted infections once economic conditions and income inequality are taken into account. Nor should the finding that countries in which more people would not want a person with AIDS as a neighbor have lower estimated levels of these infections be read as evidence that stigma protects against HIV. One interpretation consistent with our findings and with earlier research is that fear of rejection may discourage people from getting tested, disclosing their status, and seeking care, so that infections become less visible to health systems. In this sense, stigma may shape not only who can reach care but also who is counted. Because these patterns were observed between countries, and weakened considerably when comparisons were made within world regions, they should be interpreted as features of national and regional contexts and not as evidence about individual behavior. From a policy perspective, rights-based stigma reduction integrated with confidential testing and early care may improve access to services; where testing expands, short-term increases in detected cases should not automatically be interpreted as epidemiological deterioration.

Using a harmonized cross-national dataset, we used blockwise modeling, reduced multicollinearity through component extraction, applied robust standard errors, and tested for indirect effects using a bootstrap-based indirect-effect decomposition. These choices strengthened inference under distributional asymmetry and helped reveal underlying structural patterns. Nonetheless, an ecological, secondary-data, cross-sectional design imposes important constraints: it is not capable of causal identification; because the country is the unit of analysis, these associations are susceptible to ecological fallacy and should not be interpreted as individual-level effects; residual confounding and cross-national measurement heterogeneity remain possible; and descriptive indirect/total ratios become unstable when total associations are weak. Data harmonization also entails unavoidable methodological compromises. The stigma measure is likewise limited: it is a single general-population social-distance item and does not capture the anticipated, internalized, enacted, and intersectional dimensions indexed by validated instruments such as the Berger HIV Stigma Scale or the PLHIV Stigma Index, which are not available cross-nationally. The dimensions most proximal to individual testing and disclosure behavior are therefore not directly observed, and the reported associations may not generalize to them. The religiosity measure is subject to an analogous constraint. The component was derived from the subjective importance of religion and belief in God and therefore indexes religious salience and belief, only two dimensions of a broader multidimensional construct [48], as reflected in established religiosity frameworks such as the Centrality of Religiosity Scale [49] and the Duke University Religion Index [50]. Religious practice, ritual participation, private devotion, religious identity, and religiously grounded sexual morality were not represented in the analytic component. In addition, religion-related family items (D003 and D031) were excluded because of very high missingness. The null findings should therefore be interpreted as applying specifically to religious salience and belief as operationalized here, rather than as evidence that religiosity in its broader multidimensional sense is unrelated to HIV or non-HIV STI burden. Although PCA reduced severe multicollinearity and retained more than 90% of the variance in each two-item component, component scores emphasize shared variance and may obscure item-specific associations among the underlying cultural indicators. The outcome measures are themselves model-based estimates: GBD HIV prevalence and non-HIV STI incidence are produced by estimation models that, for many low- and middle-income countries, incorporate covariates and assumptions about testing and detection, so the observed associations may partly reflect features of these estimation procedures, and we could not restrict the analysis to countries with directly measured, nationally representative HIV prevalence. Consistent with a possible ascertainment contribution, adjusting for ART coverage (a proxy for detection completeness, available for 55 countries) attenuated the inverse social-distancing–HIV association by roughly 13–34% without eliminating it (Section 3; Supplementary Table S8), so we cannot fully separate a genuinely lower burden from less complete case detection in higher-social-distancing settings. The non-HIV STI outcome also aggregates pathogens (e.g., chlamydia, gonorrhea, syphilis, and trichomoniasis) with markedly different transmission dynamics, surveillance systems, and asymptomatic fractions, which may obscure pathogen-specific patterns that a stratified analysis—beyond the available data—could reveal. The sample, shaped by World Values Survey coverage, also underrepresents Sub-Saharan Africa (9 of 68 countries), limiting generalizability to the highest-burden settings; consistent with this, a region fixed-effect specification attenuated the social-distancing association (see Section 3); the social-distancing–burden association should therefore be read primarily as a between-region ecological signal rather than as robust within-region evidence. In addition, we deliberately conducted unweighted analyses to enhance comparability across countries, a decision with trade-offs—notably a degree of loss of information on variance structure. Finally, combining a modest sample with several analytic choices entails researcher degrees of freedom; although we distinguish a primary analysis from secondary and sensitivity analyses, we did not perform formal cross-validation, and estimates should be read with appropriate caution. Future work could mitigate these limitations through multilevel frameworks, comprehensive sensitivity summaries, and longitudinal or quasi-causal designs.

We recommend the following measures:

- Pair stigma-reduction campaigns with extended, confidential testing and provider training and anticipate that increased diagnoses in early phases may reflect successful ascertainment rather than rising transmission.
- Incorporate stigma trackers, testing coverage, positivity, and time-to-treatment into routine dashboards and not mere prevalence.
- In settings with high inequality and HIV burden, concentrate on community-led outreach and linkage-to-care resources and employ anti-discrimination enforcement to protect key populations.
- Implement stigma reduction, confidential testing, linkage to care, condom access, PrEP delivery, and clinician training as complementary rather than sequential strategies.
- Note that reductions in social distancing may initially be accompanied by increases in recorded diagnoses where testing coverage and disclosure improve; align funding and performance metrics to avoid penalizing programs for better detection.
- Align health initiatives with education and labor policies to address structural vulnerability that amplifies HIV in unequal settings.

## Figures and Tables

**Figure 1:**
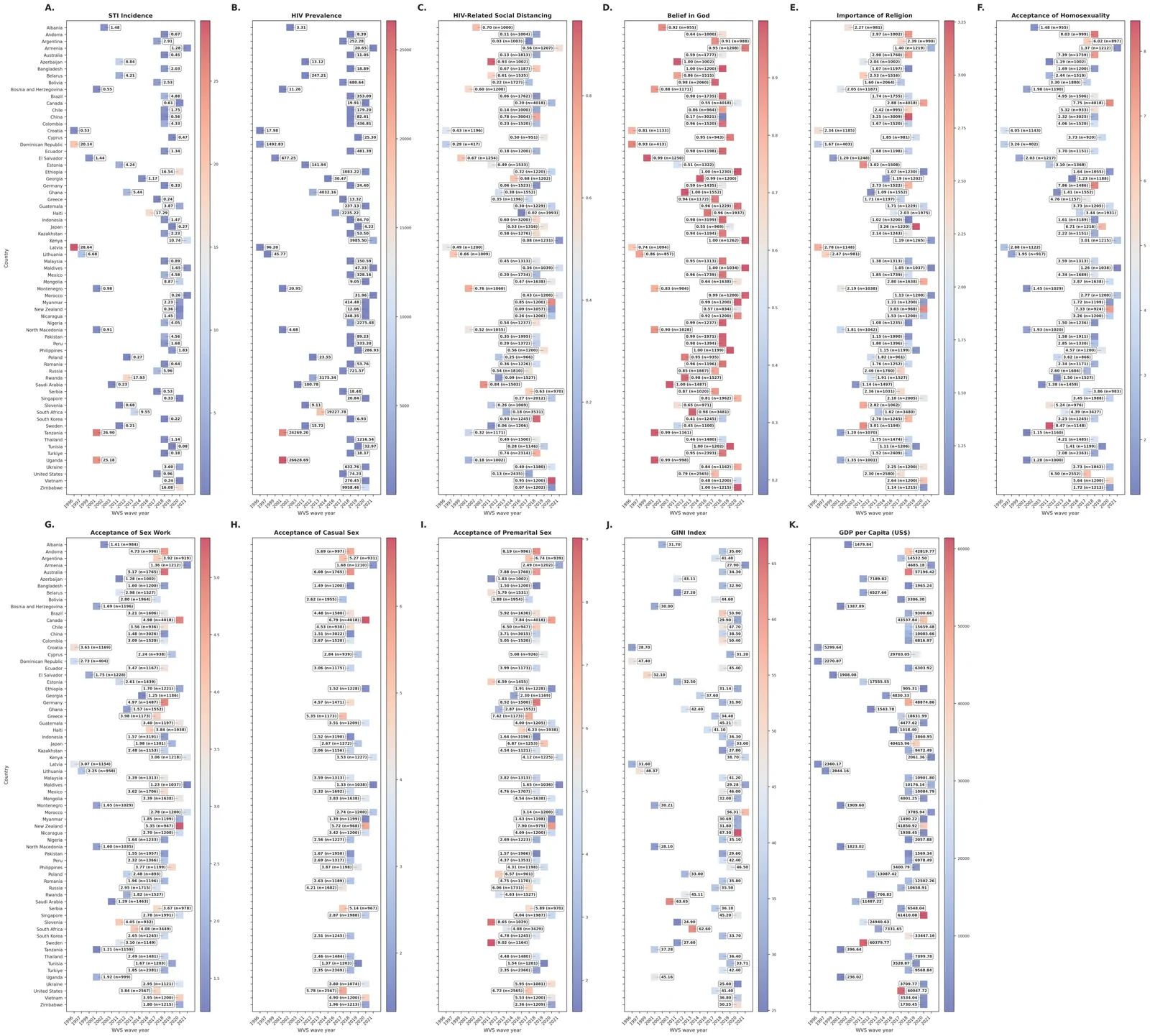
Cross-sectional distribution of study variables across 68 countries. Panels show country-level means or proportions from each country’s selected WVS wave together with HIV prevalence, non-HIV STI incidence, GDP per capita, and GINI: (**A**) non-HIV STI incidence (age-standardized rate per 100,000); (**B**) HIV prevalence (per 100,000); (**C**) HIV-related social distancing (proportion who would not want people with AIDS as neighbors); (**D**) belief in God (proportion); (**E**) importance of religion (original coding: 1 = very important to 4 = not at all important); (**F**) acceptance of homosexuality; (**G**) acceptance of sex work; (**H**) acceptance of casual sex; (**I**) acceptance of premarital sex; (**J**) GINI index; (**K**) GDP per capita (current US$). For (**F**–**I**), 1 = never justifiable and 10 = always justifiable. Colors indicate the magnitude of each variable; labels show country-level values and, for WVS items, the number of respondents (n). Country-level epidemiological and economic values are reported in Supplementary Table S6.

**Figure 2:**
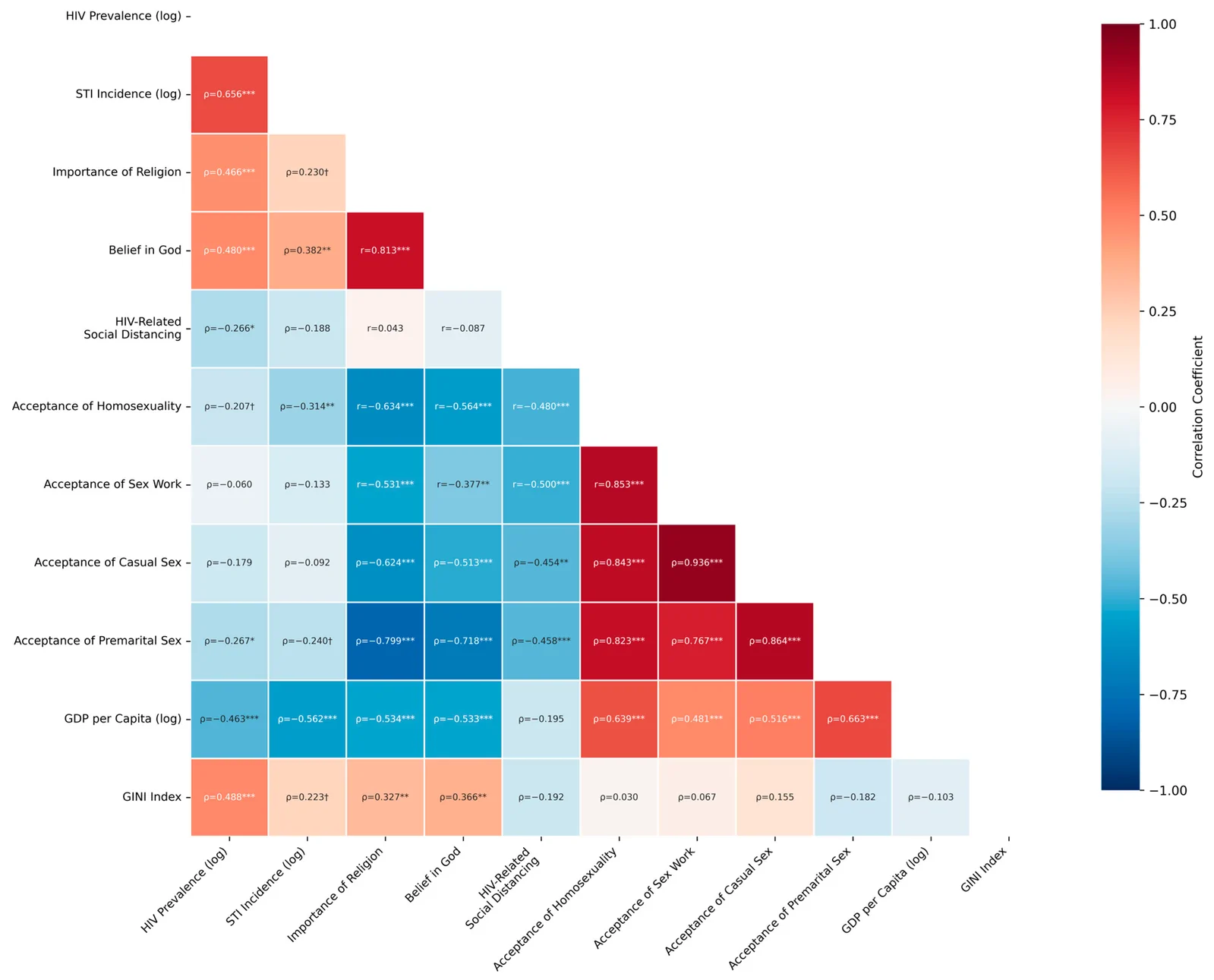
Pairwise correlations among country-level study variables. Pearson (r) or Spearman (ρ) coefficients are shown according to the distributional form used in the analysis; symbols denote statistical significance (†: *p* < 0.10; *: *p* < 0.05; **: *p* < 0.01; ***: *p* < 0.001). HIV prevalence, STI incidence, and GDP per capita are displayed on the transformed scales indicated in the figure.

**Figure 3:**
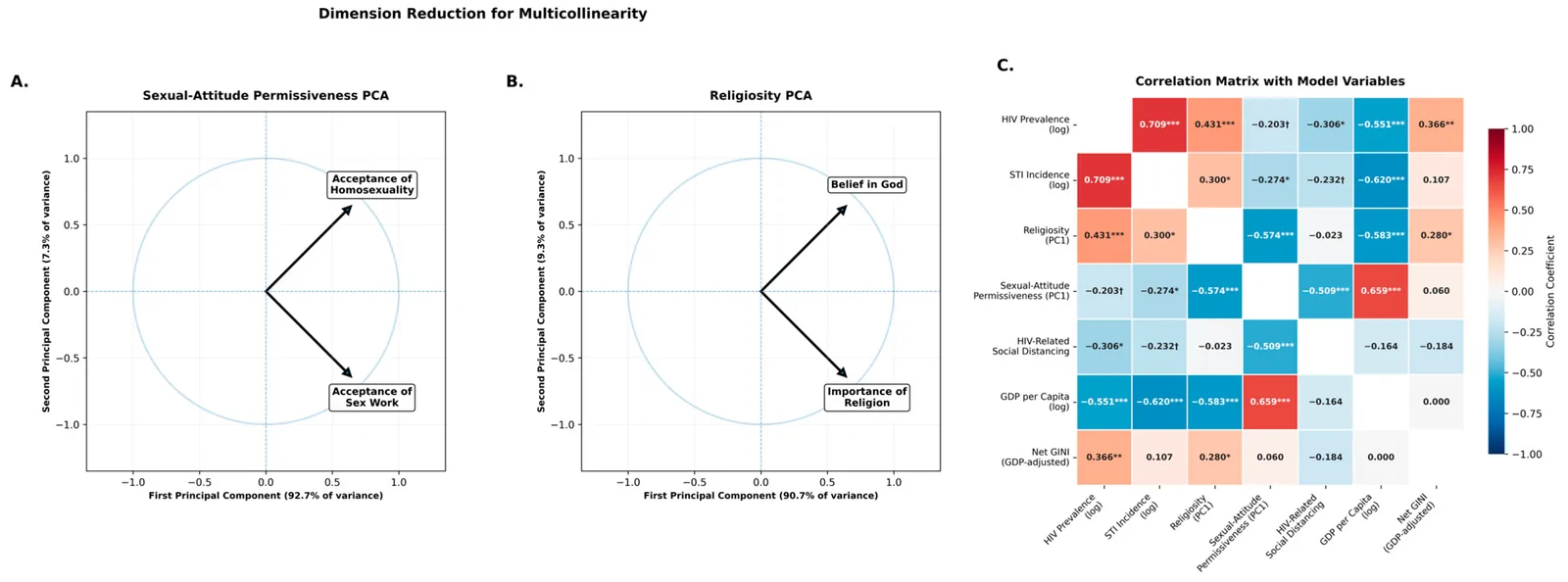
PCA dimension reduction and correlations among model variables. (**A**) Loadings for Acceptance of Homosexuality and Acceptance of Sex Work on Sexual-Attitude Permissiveness PC1 (92.7% variance explained); (**B**) loadings for Importance of Religion and Belief in God on Religiosity PC1, representing religious salience and belief (90.7%); (**C**) correlation matrix among transformed/component variables used in the hierarchical models. In (**C**), symbols denote statistical significance (†: *p* < 0.10; *: *p* < 0.05; **: *p* < 0.01; ***: *p* < 0.001).

**Figure 4:**
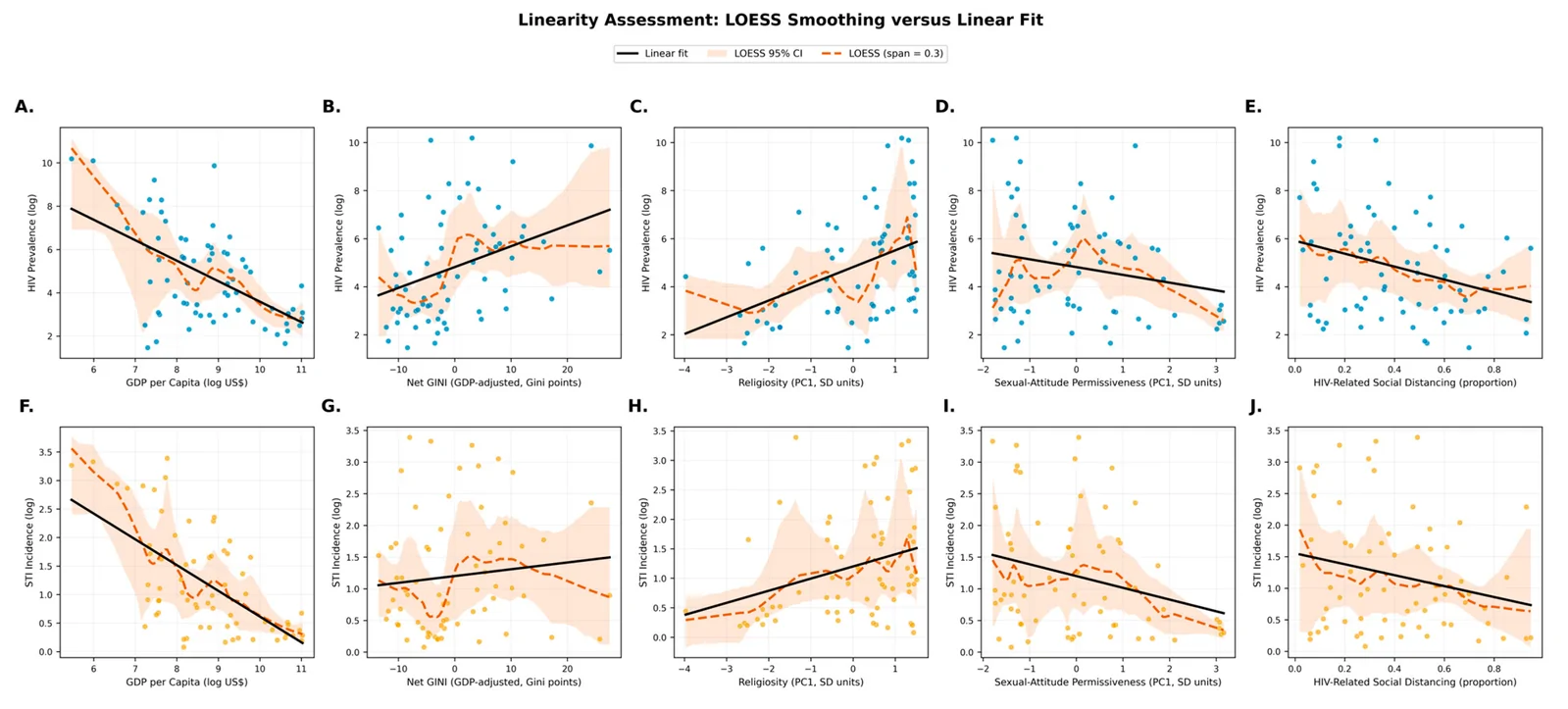
Assessment of linear functional form for HIV (**A**–**E**) and non-HIV STI (**F**–**J**) outcomes. Each panel compares the linear fit with LOESS smoothing and its 95% confidence band for log GDP, Net GINI, Religiosity PC1, Sexual-Attitude Permissiveness PC1, and HIV-related social distancing. Each point represents one country (blue: log HIV prevalence, (**A**–**E**); orange: log non-HIV STI incidence, (**F**–**J**)). The panels serve as visual diagnostics alongside AIC/BIC comparisons of alternative functional forms.

**Table 1:** Study Variables, Data Sources, and Analytical Roles.

Variable Name	Type	Source & Description
HIV prevalence (ages 15–49, per 100,000 population)	Dependent variable (continuous)	GBD 2021 estimates from the Institute for Health Metrics and Evaluation (IHME). High values indicate a large proportion of people living with HIV
STI incidence (ages 15–49, age-standardized rate per 100,000 population)	Dependent variable (continuous)	GBD 2021 estimates from IHME; age-standardized incidence rate of sexually transmitted infections excluding HIV, per 100,000 population (e.g., chlamydia, gonorrhea, and syphilis). High values indicate heavier infectious disease burden and greater transmission risk
GDP per capita ($)	Control (continuous)	World Development Indicators (WDI) of the World Bank—GDP per capita (current US$)
GINI Index	Control input for Net GINI	World Bank WDI—Income Inequality Index (0 = perfect equality, 100 = maximum inequality). High values indicate greater income inequality
GINI index (for missing observations)	Control input for Net GINI	Compiled by authors from reliable sources (e.g., International Monetary Fund and World Inequality Database) via Zenodo; closest available year.
F118—Homosexuality (attitude)	Independent (component source; multivariable model)	World Values Survey (WVS): Acceptance of homosexuality
F119—Sex work (attitude)	Independent (component source; multivariable model)	WVS: Acceptance of sex work
F132—Casual sex (attitude)	Exploratory norm (bivariate only; incomplete coverage)	WVS: Acceptance of casual sex
F135A—Premarital sex (attitude)	Exploratory norm (bivariate only; incomplete coverage)	WVS: Acceptance of premarital sex
A006—Importance of religion	Independent (component source; multivariable model)	WVS: How important is religion in your life?
A124_07—Neighbor with AIDS	Independent (social-distance item; multivariable model)	WVS: Would not like to have people with AIDS as neighbors
F050—Belief in God	Independent (component source; multivariable model)	WVS: “Do you believe in God?”

**Table 2:** Associations of Economic, Normative–Cultural, and HIV-Related Social-Distancing Blocks with HIV and STI Burden.

Variables with Sequential Model Numbers	DV Models	1: Economic Structure Block		2: Normative–Cultural Block		3: HIV-Related Social-Distancing Block	
		*b* (SE) [95% CI]	*p*	*b* (SE) [95% CI]	*p*	*b* (SE) [95% CI]	*p*
1: log(GDP)	log(HIV)	**−0.946 (0.167) [−1.273, −0.618]**	<0.001 ***	**−1.157 (0.208) [−1.564, −0.749]**	<0.001 ***	**−1.063 (0.209) [−1.473, −0.654]**	<0.001 ***
	log(STI)	**−0.452 (0.064) [−0.577, −0.326]**	<0.001 ***	**−0.583 (0.118) [−0.814, −0.352]**	<0.001 ***	**−0.537 (0.095) [−0.722, −0.351]**	<0.001 ***
1: GINI (GDP-adjusted)	log(HIV)	**0.087 (0.031) [0.026, 0.147]**	0.005 **	**0.075 (0.031) [0.015, 0.135]**	0.014 *	**0.074 (0.030) [0.015, 0.132]**	0.014 *
	log(STI)	0.011 (0.013) [−0.014, 0.036]	0.400	0.012 (0.013) [−0.014, 0.038]	0.361	0.011 (0.013) [−0.014, 0.037]	0.381
+2: Religiosity C.	log(HIV)	–	–	0.187 (0.232) [−0.268, 0.641]	0.421	−0.055 (0.208) [−0.463, 0.353]	0.791
	log(STI)	–	–	−0.059 (0.107) [−0.268, 0.150]	0.579	−0.179 (0.109) [−0.392, 0.035]	0.101
+2: Sexual-Attitude Permissiveness C.	log(HIV)	–	–	**0.461 (0.187) [0.094, 0.828]**	0.014 *	−0.025 (0.250) [−0.515, 0.466]	0.922
	log(STI)	–	–	0.133 (0.095) [−0.053, 0.319]	0.160	−0.107 (0.106) [−0.315, 0.101]	0.314
+3: HIV-related social distancing	log(HIV)	–	–	–	–	**−3.157 (1.033) [−5.181, −1.133]**	0.002 **
	log(STI)	–	–	–	–	**−1.561 (0.428) [−2.399, −0.723]**	<0.001 ***
R^2^	log(HIV)	0.438		0.479		0.554	
	log(STI)	0.396		0.429		0.531	
Adjusted R^2^	log(HIV)	0.421		0.446		0.518	
	log(STI)	0.378		0.393		0.493	
ΔR^2^	log(HIV)	–		0.041 (1–2)		0.075 (2–3)	
	log(STI)	–		0.033 (1–2)		0.102 (2–3)	
Wald’s χ^2^/*p*	log(HIV)	–		7.08/0.029 * (1–2)		9.34/0.002 ** (2–3)	
	log(STI)	–		2.80/0.247 (1–2)		13.32/<0.001 *** (2–3)	
AIC/BIC	log(HIV)	265.4/272.1		264.3/275.4		255.8/269.1	
	log(STI)	153.7/160.4		153.9/165.0		142.5/155.9	

Unstandardized regression coefficients (*b*) are reported with HC3 heteroskedasticity-consistent standard errors in parentheses; confidence intervals and *p* values are based on HC3 heteroskedasticity-consistent covariance estimates. HIV prevalence, STI incidence, and GDP per capita were log-transformed. Net GINI represents the residual from regressing GINI on log GDP per capita. The HIV-related social-distancing indicator is the country-level proportion reporting that they would not want a person with AIDS as a neighbor. Incremental explanatory power was moderate for HIV models (Δ*R*^2^ = 0.04–0.08) and stronger for STI models (Δ*R*^2^ = 0.10), which indicates that while GDP consistently remained the dominant predictor, the addition of HIV-related social distancing improved model fit. Values in bold indicate statistical significance at *p* < 0.05. *: *p* < 0.05; **: *p* < 0.01; ***: *p* < 0.001. Abbreviations: DV, dependent variable; R^2^, coefficient of determination; AIC, Akaike information criterion; BIC, Bayesian information criterion.

**Table 3:** Exploratory indirect-effect decomposition involving sexual-attitude permissiveness, HIV-related social distancing, and HIV/STI burden.

Effect Path	DV: HIV(*log*)			DV: STI(*log*)		
	*b* (SE)	*p*	95% CI	*b* (SE)	*p*	95% CI
Sexual-attitude permissiveness → HIV-related social distancing	–0.126 (0.035)	<0.001 ***	[–0.195, –0.057]	–0.126 (0.035)	<0.001 ***	[–0.195, –0.057]
HIV-related social distancing → DV (HIV or STI)	–3.063 (1.018)	0.003 **	[–5.058, –1.068]	–1.255 (0.438)	0.004 **	[–2.113, –0.397]
Direct effect (c′)	0.007 (0.226)	0.974	[–0.436, 0.450]	–0.003 (0.093)	0.974	[–0.185, 0.179]
Indirect effect	0.386 (0.167)	0.022 *	[0.058, 0.714]	0.158 (0.071)	0.026 *	[0.020, 0.296]
Total effect (c)	0.393 (0.142)	0.006 **	[0.115, 0.671]	0.155 (0.089)	0.083	[–0.019, 0.329]
Proportion mediated (%) †	reported in Supplementary Material †			not reported		
Sobel test	z = 2.296, *p* = 0.022 *			z = 2.231, *p* = 0.026 *		

Controlling for GDP and GINI (GDP-Adjusted); DV: Dependent Variable. The descriptive indirect/total ratio is not reported for STI because the total effect is near zero, which renders it unstable. † The descriptive indirect/total ratio is reported for HIV in the Supplementary Material and, because the total association is modest and the design is cross-sectional and ecological, should not be interpreted as a causal mediation measure. These estimates describe an exploratory product-of-coefficients pattern and do not establish causal mediation because the study is cross-sectional and ecological and does not establish temporal ordering or sequential ignorability. HIV-related social distancing denotes a single general-population social-distancing item (unwillingness to have a person with AIDS as a neighbor). *: *p* < 0.05; **: *p* < 0.01; ***: *p* < 0.001.

## Data Availability

The original data presented in the study are openly available in Zenodo at https://doi.org/10.5281/zenodo.16996209. These data were derived from the following resources available in the public domain: World Values Survey; IHME Global Burden of Disease 2021; World Bank World Development Indicators; UNAIDS.

## Supplementary Materials

The following supporting information can be downloaded at: https://www.mdpi.com/article/10.3390/healthcare14213557/s1. Figure S1: Distribution of Key Social Norms and Attitudes by Country–Year (1996–2021); Table S1: Descriptive Statistics of Basic Social Norms and Attitudes Prior to Missing Data Analysis; Table S2: Last Observation Carried Backward (LOCB): Temporal Transfer Requirements by Variable; Table S3: Variables Excluded From Analysis Due to Critical Missingness; Table S4: Alternative Sources of GINI Coefficient for Countries With Missing Data From the World Bank; Table S5: Sample distribution and disease burden by World Bank region (n = 68); Table S6: Country-level values: WVS wave year, region, and study indicators (n = 68); Table S7: Sensitivity analyses for HIV and STI models (HC3 regression coefficients); Table S8: Ascertainment sensitivity: adjusting the social-distancing → HIV association for ART coverage; Table S9: Decomposition of the association between sexual-attitude permissiveness and epidemiological outcomes through HIV-related social distancing (exploratory, product-of-coefficients).
