# Supplementary material for: Sexual Norms, Religiosity, and Stigma in the Epidemiology of HIV and STI: A Cross-National Ecological Study of 68 Countries
Source: Healthcare (Basel). 2026 Oct 22;14(21):3557. doi: 10.3390/healthcare14213557 (PMC13763371; doi:10.3390/healthcare14213557)
Supplement: Supplementary file 1 [file healthcare-14-03557-s001.zip › healthcare-4527054-supplementary.pdf]

Supplementary Data Sheet S1

1 Details of Data Preprocessing and Analysis of Initial Missing Data

The source WVS file contained 443,488 distinct respondents. Twenty-nine records in which all 11 selected variables were coded with negative WVS missing-value codes were excluded, leaving 443,459 respondents. No duplicate respondent identifiers remained. These negative codes follow WVS conventions (−5 = missing, −4 = not asked, −3 = not applicable, −2 = no answer, −1 = don't know) and were treated as missing at the item level. The cleaned source WVS dataset comprised 443,459 respondents from 108 countries and 296 country–year combinations (survey waves 1981–2022), rather than the final analytical cross-section of 68 countries. These respondents contributed 3,070,950 valid item-level responses across the selected variables before country–year aggregation.

Another variable used in the study is the GINI index series. When integrated on a country–year basis, only 140 out of 307 observations contained values in the first stage, while 167 observations remained missing, and completion rate remained at 45.6%. To address the missing data, values from the nearest year were assigned for each country; in cases of equal distance, the more recent year was preferred. As a result, the number of complete values increased to 213, whereas the number of missing observations decreased to 94, and the completion rate reached 69.4%. Thus, the GINI series became usable with broader coverage in analyses, although gaps in the series warrant caution in interpreting the results.

The study also intended to include condom use rate in high-risk sex as a variable. These data are compiled by UNAIDS and the Bill & Melinda Gates Foundation (UNAIDS 2024). However, when integrated into the dataset on a country–year basis, coverage remained extremely limited. Despite initial matching and nearest year value assignments, values were identified for only 11 country–year observations, while 296 observations remained missing, and the completion rate reached only 3.6%. Additionally, an average difference of 16 years (min: 5, max: 28 years) between years to which available values were assigned and analysis years prevented the reliable use of the variable. For these reasons, the condom use indicator was excluded.

Table S1. Descriptive Statistics of Basic Social Norms and Attitudes Prior to Missing Data Analysis

Variable	Country–Year Count	Total Observations	$\mu \pm \sigma$	Min–Max	Median (Q_1–Q_3)
Importance of religion	296	420,800	1,969 ± 0,677	1,013–3,724	1,838 (1,363–2,525)
Neighbor with AIDS	286	407,377	0,435 ± 0,263	0,000–0,995	0,426 (0,199–0,661)
Acceptance of homosexuality	292	396,143	3,378 ± 1,872	1,005–9,031	2,89 (1,810–4,508)
Acceptance of sex work	272	377,219	2,734 ± 1,08	1,017–6,228	2,538 (1,948–3,391)
Acceptance of casual sex	62	89,470	3,499 ± 1,589	1,065–7,179	3,328 (2,223–4,563)
Acceptance of premarital sex	116	169,478	4,625 ± 2,172	1,116–9,020	4,632 (2,49–6,225)
Belief in God	223	314,839	0,848 ± 0,192	0,175–1,000	0,946 (0,771–0,987)
Religious sharing with spouse	11	12,052	0,673 ± 0,162	0,415–0,936	0,716 (0,559–0,743)
Importance of religious belief in marriage	12	17,228	2,073 ± 0,426	1,269–2,801	2,161 (1,75–2,271)

Descriptive summary statistics prior to missing data analysis show country–year averages for variables as follows: Importance of religion (mean = 1.969, SD = 0.677), Neighbor with AIDS (mean = 0.435, SD = 0.263), Acceptance of homosexuality (mean = 3.378, SD = 1.872), Acceptance of prostitution

(mean = 2.734, SD = 1.080), Acceptance of casual sex (mean = 3.499, SD = 1.589), Acceptance of premarital sex (mean = 4.625, SD = 2.172), and Belief in God (mean = 0.848, SD = 0.192). Additionally, the number of country–year combinations varies across variables due to differences in coverage. For example, 296, 286, 292, 272, 62, 116, 223, 11, and 12 country–year combinations were identified for Importance of religion, Neighbors with AIDS, Acceptance of homosexuality, Acceptance of prostitution, Acceptance of casual sex, Acceptance of premarital sex, Belief in God, Religious sharing with spouse, and Importance of religious belief in marriage, respectively (Table S1).

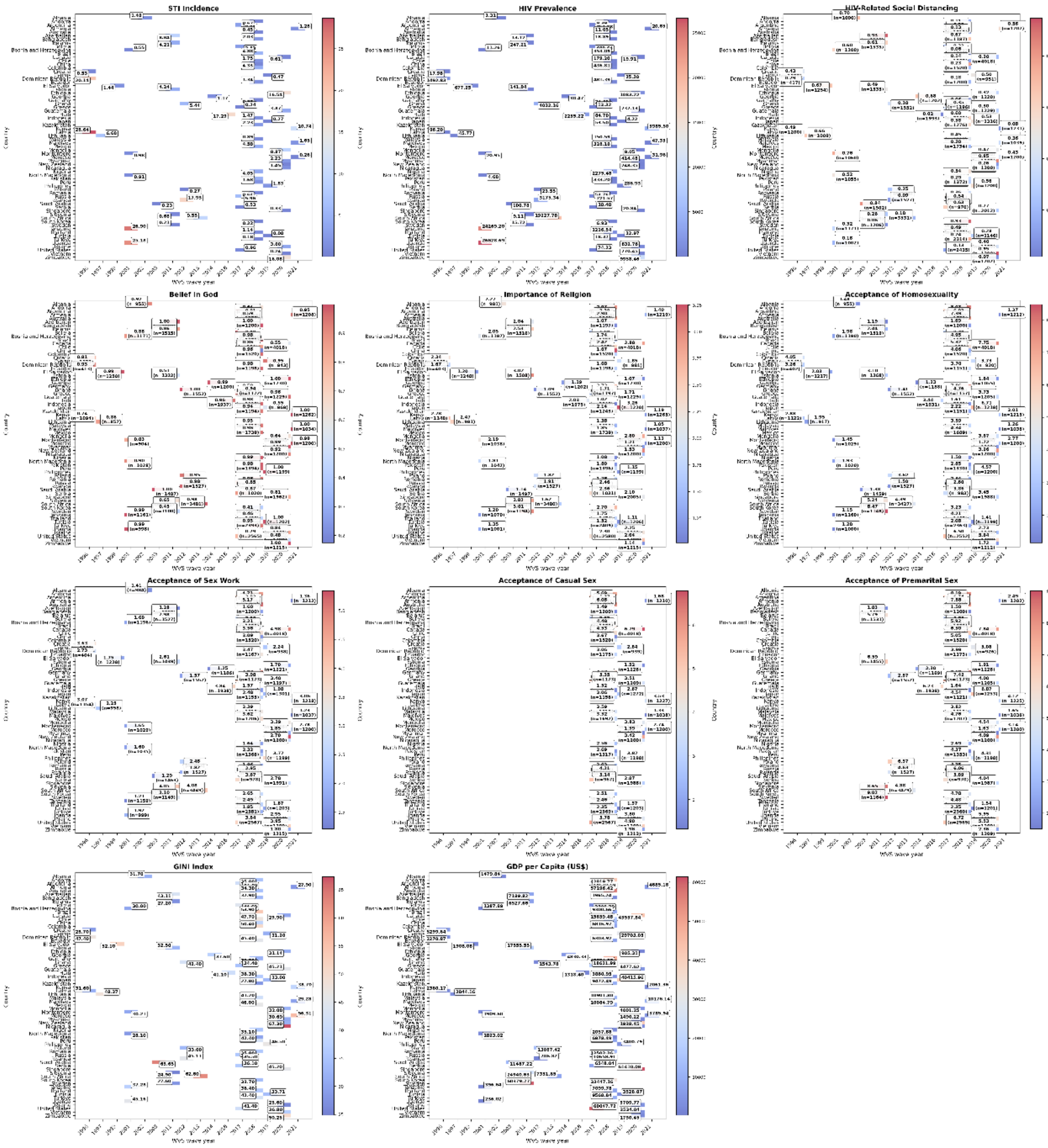

Figure S1. Distribution of Key Social Norms and Attitudes by Country–Year (1996–2021)

When examining the distribution of basic social norms and attitudes by country between 1996 and 2021 given the direction of scales and lower–upper values, the following results emerge: values for STI burden (4.490 ± 6.764 [0.081–28.638]) and HIV prevalence (1,585.716 ± 4,972.424 [3.312–26,628.694]) exhibit substantial differences in health indicators across countries. Rejection of neighbors with AIDS (0.407 ± 0.249 [0.019–0.947; n = 98,194]), HIV-related social distancing, defined

as the country-level proportion of respondents reporting that they would not want a person with AIDS as a neighbor (a single-item social-distance indicator rather than a comprehensive stigma scale, where higher values indicate greater social distancing toward people living with HIV/AIDS), indicates that an average of 41% of participants avoid being neighbors with patients with AIDS. Among the religion-related variables, Belief in God (0.853 ± 0.190 [0.175–0.999; n = 96,581]) reveals that 85% of the participants believe in God, while Importance of religion (1.933 ± 0.659 [1.020–3.256; n = 98,266]) reveals that many countries consider religion to be relatively important. Regarding sexual norm variables, values for Acceptance of homosexuality (3.389 ± 1.955 [1.153–8.470; n = 95,816]), Acceptance of prostitution (2.708 ± 1.090 [1.211–5.346; n = 96,868]), and Acceptance of casual sex (3.368 ± 1.465 [1.328–6.789]; n = 67,289, 45 countries) demonstrate that these behaviors are evaluated as close to *never justifiable* in the majority of countries. In contrast, Acceptance of premarital sex (4.751 ± 2.102 [1.505–9.020]; n = 84,088, 56 countries) produces a relatively higher average, which indicates that social acceptance of this behavior is more widespread compared with other sexual norms. Among the economic indicators, GINI index reached 38.589 ± 9.362 (24.900–67.300), while GDP per capita was USD 12,727.213 ± 16,582.105 (236.021–61,410.079). In summary, when examining minimum and maximum values, major differences were observed across countries in terms of sexual health indicators, social attitudes, religious commitment, sexual norms, and economic factors (Figure S1).

2 Assessment of Systematic Missingness

Missing data patterns in the dataset denote partially systematic characteristics. Two variables, namely, D003 (Religious sharing with spouse, i.e., sharing religious views between spouses) and D031 (Importance of religious belief in marriage, i.e., consideration of important religious beliefs in marriage), contain very high missingness (approximately 98%). This finding indicates that these questions may have been added to the WVS in later waves or were only asked during certain waves. Missing rates for other variables ranged from 0.9% to 42.6%. Missingness is particularly higher for sexuality-themed questions (average rate of acceptance of casual sex: 42.6%, average rate of acceptance of premarital sex: 27.8%). In contrast, missing rates are relatively low for health and economic indicators (9%–10%). A year-based examination illustrates that missingness relatively decreased during the 2017–2021 period (13%–20%), whereas it was higher in the pre-2014 period (28%–42%). Additionally, perfect correlations (r = 1.000) indicate that a number of variables are missing from the same observations, while moderate–high correlations (0.7 and above) indicate that questions on sexuality topics in particular were left unanswered in similar ways.

The high missing rates for casual sex acceptance index (42.6%) and premarital sex rate variables (27.8%) and their systematic nature demonstrate that these questions were left unanswered due to social sensitivities. In this case, the last observation carried backward (LOCB) implementation was evaluated, and it was deemed that this approach may introduce systematic bias by carrying presumably more conservative attitudes from past periods to the present. Additionally, the initial 41.7% missing rate in the GINI coefficient reflects difficulty in data collection for economic indicators, which raises doubt on the reliability of backward carrying for such rapidly changing indicators. For these reasons, LOCB was initially applied to assess data integrity; variant attitudes and behaviors were then evaluated according to missingness in the first stage.

3 Trial Application of Last Observation Carried Backward

The feasibility and appropriateness of using the LOCB methodology for the dataset were systematically examined. LOCB involves imputing missing values in the most recent observation period by carrying backward the most recent available historical data for each country. Although this approach could potentially increase the sample size from 68 to 88 countries, the temporal validity of this imputation requires careful scrutiny, particularly for variables that represent rapidly evolving social attitudes and economic indicators (Table S2).

Table S2. Last Observation Carried Backward (LOCB): Temporal Transfer Requirements by Variable

Variable	Countries Requiring LOCB	Minimum Year Gap	Maximum Year Gap	Mean Year Gap	Median Year Gap	Countries with 10+ Year Gap
**Attitudes And Values**						
Acceptance of homosexuality	2	11	17	14.0	14	2 *(100%)*
Acceptance of sex work	6	4	17	10.2	12	4 *(67%)*
Belief in God	7	4	17	10.3	11	4 *(57%)*
**Health Indicators**						
STI incidence	7	8	24	13.1	11	6 *(86%)*
HIV prevalence	7	8	24	13.1	11	6 *(86%)*
**Economic Indicator**						
GDP per capita *(current $)*	1	21	21	21.0	21	1 *(100%)*

The most extreme cases of temporal transfer requirements warrant particular attention. The Czech Republic will require 24-year-old data (since 1998) for HIV and STI indicators, thus representing health statistics recorded prior to major advances in antiretroviral therapy (ART) and prevention programs. Venezuela's GDP per capita will need to be carried forward from 2000, which predates the country's economic crisis and hyperinflation period. Egypt will require 17-year-old data for acceptance of homosexuality and sex work and Belief in God, spanning a period that includes the Arab Spring and subsequent social transformations (Table S2).

The temporal gaps identified present three major methodological concerns that ultimately led to the decision to forgo LOCB implementation:

- Variables related to social attitude provide evidence of rapid liberalization in many countries. Variables for measuring acceptance of homosexuality and sex work, which would require 10+ year transfers in 67%–100% of affected countries, are particularly susceptible to cohort effects and changes in generational value. Carrying backward conservative attitudes from the early 2000s to represent current populations would introduce systematic downward bias, which would be particularly problematic given the theoretical importance of these variables in explaining sexual health outcomes.
- Health indicators demonstrate substantial temporal evolution. The requirement to use data on HIV and STI from up to the last 24 years for a number of countries overlooks fundamental epidemiological transitions. The case of the Czech Republic exemplifies this issue. Using HIV prevalence data for 1998 would predate its successful harm reduction programs and widespread availability of ART, which renders these historical data fundamentally incomparable with contemporary observations.

- Economic indicator (GDP per capita) exhibits high volatility over extended periods. For example, Venezuela's requirement for 21-year-old GDP data spans multiple economic crises, currency devaluations, and structural economic changes. These dramatic economic transformations render historical values meaningless for contemporary analysis.

Given these temporal validity concerns, analysis proceeded without LOCB implementation, thus accepting a trade-off of reduced sample size (n = 68) for enhanced data integrity. The 20 countries excluded due to missing contemporary data represent a diverse geographic and economic spectrum, including developed (the United Kingdom, the Netherlands, Switzerland, and Norway) and developing (Egypt, India, and Uzbekistan) nations. While this exclusion may limit generalizability, it prevents the introduction of systematic temporal bias, which would be particularly problematic given the study's focus on contemporary relationships among social attitudes, economic conditions, and sexual health outcomes.

The final analytical sample of 68 countries provides complete contemporary data across the key variables, thus ensuring temporal consistency and avoiding the assumption of stability in inherently dynamic social and economic phenomena. This methodological decision prioritizes internal validity over sample size by recognizing that meaningful inference requires temporally coherent observations instead of artificially inflated samples based on questionable imputation assumptions.

4 Observation and Variable Cleaning

Following the decision to forgo LOCB implementation due to concerns about temporal validity, the most recent year's observations for each country were employed without imputing historical data. This approach resulted in a cross-sectional dataset of 68 countries providing data on 13 key variables after excluding those with critical missingness. The final cross-sectional dataset used each country's most recent eligible WVS wave, with survey years ranging from 1996 to 2021 and a median survey year of 2018. Of the 68 countries, 46 contributed a WVS wave from 2015 or later. Epidemiological and economic indicators were linked to the corresponding country–year where available.

The initial assessment revealed that variables related to spousal religious beliefs (D003 [Religious belief sharing with spouse] and D031 [Importance of religious beliefs in marriage]) exhibited systematic missingness (nearly 90%), which implies these questions were added in later WVS waves or administered only in specific regional modules. Additionally, the indicator condom use at last high-risk sex, derived from UNAIDS, could not be matched with the country sample. These three variables were consequently excluded from all analyses (Table S3).

Table S3. Variables Excluded From Analysis Due to Critical Missingness

Variable	Type	Source & Description
Condom use at last high-risk sex (ages 15–49, %)	Independent (continuous)	UNAIDS and BMGF indicator compiled by Our World in Data
D003 (Religious belief sharing with spouse)	Independent (family/relationship value)	WVS: "Sharing religious beliefs with spouse"
D031 (Importance of religious beliefs in marriage)	Independent (family/relationship value)	WVS: "Importance of religious beliefs in marriage"

These variables were excluded, because their missingness exceeded 85%, thus preventing reliable cross-country analysis.

After the exclusion of these variables, the missing data structure in the sample demonstrated a clear bifurcation. Data for 10 core variables, namely, importance of religion, happiness, self-rated health status, HIV-related social distancing, acceptance of homosexuality, acceptance of sex work, Belief in God, HIV prevalence, STI incidence, and GDP per capita, were complete for the 68 countries (0% missingness). The availability of complete data for the primary dependent variables and key theoretical predictors provides a robust foundation for the main analyses.

In contrast, three additional variables exhibited moderate to high missingness: acceptance of casual sex (33.8% missingness, n = 45), GINI coefficient (33.8% missingness, n = 45), and acceptance of premarital sex (17.6% missingness, n = 56). The patterns of missing data reveal systematic clustering, with complete data on 32 countries (47.1%) across 13 variables, while the remaining countries exhibit five distinct patterns of missingness primarily related to sexual norm variables and measure of economic inequality.

5 Addressing GINI Coefficient Missingness Through Alternative Sources

The GINI coefficient is essential for controlling the economic effects of inequality on HIV/STI outcomes. It initially exhibited 33.8% missingness when sourced exclusively from World Bank's SI.POV.GINI indicator. To address this limitation while maintaining methodological rigor, a systematic search for alternative reliable sources was conducted. Through this process, GINI values for the 23 countries with missing data from the World Bank were successfully identified, thus enabling the inclusion of economic inequality measures for the entire sample (n = 68).

Alternative data sources comprised three primary categories. First, the World Bank Poverty and Inequality Platform provided updated or historical GINI values for 15 countries, with temporal offsets ranging from 1 to 8 years. These values maintain consistency with the World Bank methodology by using consumption-based measures for developing countries (Ethiopia, Bangladesh, Haiti, Maldives, Mongolia, Montenegro, Myanmar, Rwanda, Tanzania, Tunisia, Uganda, and Zimbabwe) and income-based measures for higher-income nations (Guatemala and Nicaragua). Second, the World Inequality Database supplied pre-tax national income GINI coefficients for six countries (i.e., Azerbaijan, Lithuania, Morocco, Saudi Arabia, and Nicaragua), which represents a different but academically recognized inequality metric. Third, national statistical offices and international organizations provided country-specific measures, including OECD standardized values for New Zealand and South Africa, IMF estimates for Andorra, and calculations by Singapore's Department of Statistics.

Heterogeneity in data sources introduces several methodological considerations. The combination of income- and consumption-based measures reflects underlying differences in data collection capacity and economic structure across countries, in which consumption-based GINI typically yields values that are 5–7 points lower than income-based measures for equivalent inequality levels. Temporal misalignment, while averaging 3.4 years, assumes relative stability in patterns of inequality over short periods, which is a reasonable assumption given GINI's slow-changing nature. However, it is potentially problematic for countries undergoing rapid economic transition. The use of pre- versus post-tax measures in a number of countries may overstate inequality relative to post-transfer distributions used in the standard calculations of the World Bank. Despite these limitations, the completed GINI dataset enables crucial analyses of the independent association between economic inequality and sexual health outcomes. The alternative sourcing strategy prioritized methodological transparency over perfect comparability. Table S4 presents all data sources and temporal adjustments.

After the successful augmentation of GINI data through alternative sources, GDP-adjusted GINI (hereafter Net GINI, consistent with the main text) was introduced by regressing GINI on log(GDP per capita) and extracting residuals. This transformation has two functions. First, it provided a clear interpretation of inequality patterns by isolating the GINI component that is independent of economic development level. Second, it addressed potential collinearity issues in regression models. The residuals capture whether a country has higher or lower inequality than expected given its GDP level, which facilitates a nuanced analysis of the association between relative inequality, instead of raw GINI values confounded with wealth differences, and HIV prevalence and STI incidence.

In the dataset, variables with higher missingness (acceptance of casual sex: 33.8% missingness, n = 45, premarital sex: 17.6% missingness, n = 56) were retained but excluded from regression analyses due to incomplete coverage. These variables were utilized only in exploratory correlation analyses to examine broad patterns of sexual-attitude permissiveness, with an appropriate acknowledgment of the decreased sample size for each pairwise correlation. The decision to exclude these variables from multivariate models was intended to avoid a substantial reduction in the sample to 32 countries, which would occur with listwise deletion. Alternatively, their inclusion in bivariate analyses provided supplementary insights into the relationships among different dimensions of sexual norms and health outcomes. All correlation coefficients involving these partially observed variables were interpreted with an explicit notation of the actual sample size for each correlation pair.

Table S4. Alternative Sources of GINI Coefficient for Countries With Missing Data From the World Bank

Country	Survey Year	GINI Value	Source and Description
Andorra	2018	35.0	International Monetary Fund, 2019 data
Azerbaijan	2011	43.11	World Inequality Database, 2011 (pre-tax national income, s coefficient, adults, equal split)
Bangladesh	2018	32.9	World Bank (2016 = 32.4, 2022 = 33.4; year-weighted average)
Croatia	1996	28.7	World Bank, 1998 data
Ethiopia	2020	31.14	World Bank (2021 data, PIP 2025), Poverty and Inequality Platform (consumption-based)
Guatemala	2020	45.21	World Bank (2023 data, PIP 2025), Poverty and Inequality Platform (income-based)
Haiti	2016	41.1	World Bank (2012 data, PIP 2025), Poverty and Inequality Platform (consumption-based)
Lithuania	1997	48.37	World Inequality Database, 1997 (pre-tax national income, GINI coefficient, adults, equal split)
Maldives	2021	29.28	World Bank (2019 data, PIP 2025), Poverty and Inequality Platform (consumption-based)
Mongolia	2020	32.08	World Bank (average of 2018 and 2022 data, PIP 2025), Poverty and Inequality Platform (consumption-based)
Montenegro	2001	30.21	World Bank (2005 data, PIP 2025), Poverty and Inequality Platform (consumption-based)
Morocco	2021	56.31	World Inequality Database, 2021 (pre-tax national income, GINI coefficient, adults, equal split)
Myanmar	2020	30.69	World Bank (2017 data, PIP 2025), Poverty and Inequality Platform (consumption-based)
New Zealand	2020	31.8	OECD, 2020 (0–1 range scaled)
Nicaragua	2020	67.3	World Inequality Database, 2020 (pre-tax national income, GINI coefficient, adults, equal split)
Rwanda	2012	45.11	World Bank (2013 data, PIP 2025), Poverty and Inequality Platform (consumption-based)

Saudi Arabia	2003	63.65	World Inequality Database, 2003 (pre-tax national income, GINI coefficient, adults, equal split)
Singapore	2020	45.2	Department of Statistics Singapore, 2020 (0–1 range scaled, before taxes)
South Africa	2013	62.6	OECD, 2015 (0–1 range scaled)
Tanzania	2001	37.28	World Bank (2000 data, PIP 2025), Poverty and Inequality Platform (consumption-based)
Tunisia	2019	33.71	World Bank (2021 data, PIP 2025), Poverty and Inequality Platform (consumption-based)
Uganda	2001	45.16	World Bank (2002 data, PIP 2025), Poverty and Inequality Platform (consumption-based)
Zimbabwe	2020	50.25	World Bank (2019 data, PIP 2025), Poverty and Inequality Platform (consumption-based)

Survey year indicates the year of WVS data collection for each country. If GINI data were unavailable for a survey year, then the closest available year was used (enclosed in parentheses). Income- and consumption-based measures are noted where applicable, given that consumption-based GINI values typically are 5–7 points lower than income-based measures for equivalent inequality levels.

The objective of this methodological strategy is to balance multiple considerations to maximize analytical rigor. The retention of all 68 countries in the primary analyses preserves statistical power, while the GDP-adjusted GINI transformation enables both economic indicators to serve as control variables without collinearity concerns. The hierarchical regression approach positions these economic variables (log GDP and GDP-adjusted GINI) as baseline controls in Model 1, thus establishing the foundational economic context. Substantive theoretical testing proceeds in two further blocks that match the main text: Model 2 adds the religiosity and sexual-attitude permissiveness components together (the Religiosity PC1 from Importance of religion and Belief in God, which explains 90.7% of their variance, and the Sexual-Attitude Permissiveness PC1 from acceptance of homosexuality and sex work, which explains 92.7% of their variance, respectively), and Model 3 adds HIV-related social distancing. This sequential model construction enables the examination of the relationship of cultural values and social attitudes with HIV/STI outcomes after accounting for economic conditions. The variance explained by each successive block reveals the relative importance of religious conservatism, HIV-related social distancing, and sexual-attitude permissiveness apart from economic determinants. By addressing GDP and inequality as necessary controls instead of primary predictors, analysis continues to focus on the theoretical relationships of interest while acknowledging that health outcomes should be understood in connection with economic context.

Supplementary Table S5 reports the sample distribution by World Bank region, Table S6 lists country-level values including the WVS wave year used for each country, Table S7 summarizes sensitivity analyses (wave-year control, 2015-or-later restriction, and region fixed effects), and Table S8 reports the ascertainment sensitivity analysis (adjustment for ART coverage).

Table S5. Sample distribution and disease burden by World Bank region (n = 68)

Region	Countries (n)	Mean HIV	Median HIV	Mean STI	Mean GDP (US$)
Sub-Saharan Africa	9	10,515.1	4,032.2	14.7	1,886
Latin America & Caribbean	13	610.4	353.1	5.2	6,530
East Asia & Pacific	13	197.7	82.4	1.5	21,438
Europe & Central Asia	25	90.9	21.0	3.0	13,952
Middle East & North Africa	3	55.3	33.0	0.2	6,267
South Asia	3	51.8	47.3	2.7	4,570
North America	2	47.0	47.0	0.8	51,793

HIV prevalence and STI incidence per 100,000; GDP per capita in current US$. Sub-Saharan Africa is markedly underrepresented relative to its share of global HIV burden.

Table S6. Country-level values: WVS wave year, region, and study indicators (n = 68). Countries are grouped by World Bank region and listed alphabetically within each region; the WVS wave year used for each country is reported in the second column.

Country	WVS wave year	Region	HIV prevalence (/100k)	STI incidence (/100k)	GDP per capita (US$)	GINI
Australia	2018	East Asia & Pacific	11.0	0.5	57196	34.3
China	2018	East Asia & Pacific	82.4	0.6	10086	38.5
Indonesia	2018	East Asia & Pacific	84.7	1.5	3861	36.3
Japan	2019	East Asia & Pacific	4.2	0.3	40416	33.0
Malaysia	2018	East Asia & Pacific	150.6	0.9	10902	41.2
Mongolia	2020	East Asia & Pacific	9.0	8.9	4001	32.1
Myanmar	2020	East Asia & Pacific	414.5	2.2	1490	30.7
New Zealand	2020	East Asia & Pacific	12.1	0.4	41851	31.8
Philippines	2019	East Asia & Pacific	286.9	1.8	3401	46.5
Singapore	2020	East Asia & Pacific	20.8	0.3	61410	45.2
South Korea	2018	East Asia & Pacific	6.9	0.2	33447	33.7
Thailand	2018	East Asia & Pacific	1216.5	1.1	7100	36.4
Vietnam	2020	East Asia & Pacific	270.4	0.2	3534	36.8
Albania	2002	Europe & Central Asia	3.3	1.5	1480	31.7
Andorra	2018	Europe & Central Asia	8.4	0.7	42820	35.0
Armenia	2021	Europe & Central Asia	20.7	1.3	4685	27.9
Azerbaijan	2011	Europe & Central Asia	13.1	8.8	7190	43.1
Belarus	2011	Europe & Central Asia	247.2	4.2	6528	27.2
Bosnia and Herzegovina	2001	Europe & Central Asia	11.3	0.6	1388	30.0
Croatia	1996	Europe & Central Asia	18.0	0.5	5300	28.7
Cyprus	2019	Europe & Central Asia	25.3	0.5	29703	31.2
Estonia	2011	Europe & Central Asia	141.9	4.2	17556	32.5
Georgia	2014	Europe & Central Asia	30.5	1.2	4830	37.6
Germany	2018	Europe & Central Asia	24.4	0.3	48875	31.9
Greece	2017	Europe & Central Asia	13.3	0.2	18632	34.4
Kazakhstan	2018	Europe & Central Asia	53.5	2.2	9472	27.8
Latvia	1996	Europe & Central Asia	96.2	28.6	2360	31.6
Lithuania	1997	Europe & Central Asia	45.8	6.7	2844	48.4
Montenegro	2001	Europe & Central Asia	21.0	1.0	1910	30.2

North Macedonia	2001	Europe & Central Asia	4.7	0.9	1823	28.1
Poland	2012	Europe & Central Asia	23.6	0.3	13087	33.0
Romania	2018	Europe & Central Asia	53.8	0.6	12502	35.8
Russia	2017	Europe & Central Asia	721.6	6.0	10659	35.5
Serbia	2017	Europe & Central Asia	18.5	0.5	6548	36.1
Slovenia	2011	Europe & Central Asia	9.1	0.7	24941	24.9
Sweden	2011	Europe & Central Asia	15.7	0.2	60380	27.6
Turkiye	2018	Europe & Central Asia	18.4	0.2	9569	42.4
Ukraine	2020	Europe & Central Asia	632.8	3.6	3710	25.6
Argentina	2017	Latin America & Caribbean	252.3	2.9	14533	41.4
Bolivia	2017	Latin America & Caribbean	680.6	2.5	3306	44.6
Brazil	2018	Latin America & Caribbean	353.1	4.9	9301	53.9
Chile	2018	Latin America & Caribbean	179.2	1.7	15659	47.7
Colombia	2018	Latin America & Caribbean	436.8	4.3	6817	50.4
Dominican Republic	1996	Latin America & Caribbean	1492.8	20.1	2271	47.4
Ecuador	2018	Latin America & Caribbean	481.4	1.3	6304	45.4
El Salvador	1999	Latin America & Caribbean	677.2	1.4	1908	52.1
Guatemala	2020	Latin America & Caribbean	237.1	3.9	4478	45.2
Haiti	2016	Latin America & Caribbean	2235.2	17.3	1318	41.1
Mexico	2018	Latin America & Caribbean	328.2	4.6	10085	46.0
Nicaragua	2020	Latin America & Caribbean	248.3	1.5	1938	67.3
Peru	2018	Latin America & Caribbean	333.2	1.7	6978	42.4
Morocco	2021	Middle East & North Africa	32.0	0.3	3786	56.3
Saudi Arabia	2003	Middle East & North Africa	100.8	0.2	11487	63.6
Tunisia	2019	Middle East & North Africa	33.0	0.1	3529	33.7
Canada	2020	North America	19.9	0.6	43538	29.9
United States	2017	North America	74.2	1.0	60048	41.4
Bangladesh	2018	South Asia	18.9	2.0	1965	32.9
Maldives	2021	South Asia	47.3	1.6	10176	29.3
Pakistan	2018	South Asia	89.2	4.6	1569	29.6
Ethiopia	2020	Sub-Saharan Africa	1083.2	16.5	905	31.1
Ghana	2012	Sub-Saharan Africa	4032.2	5.4	1544	42.4
Kenya	2021	Sub-Saharan Africa	3985.5	10.7	2061	38.7
Nigeria	2018	Sub-Saharan Africa	2275.5	4.1	2058	35.1
Rwanda	2012	Sub-Saharan Africa	3175.3	17.9	707	45.1
South Africa	2013	Sub-Saharan Africa	19227.8	9.6	7332	62.6
Tanzania	2001	Sub-Saharan Africa	24269.2	26.9	397	37.3
Uganda	2001	Sub-Saharan Africa	26628.7	25.2	236	45.2
Zimbabwe	2020	Sub-Saharan Africa	9958.5	16.1	1730	50.2

Table S7. Sensitivity analyses for HIV and STI models (HC3 regression coefficients)

**Panel A. HIV prevalence (log)**				
**Predictor**	**b**	**HC3 SE**	**95% CI**	**p**
**Reference (Model 3), n = 68**				
log GDP	−1.0632	0.2089	[−1.4726, −0.6539]	< 0.001
Net GINI	0.0735	0.0298	[0.0151, 0.1320]	0.0136
Religiosity PC1	−0.0551	0.2081	[−0.4629, 0.3527]	0.7912
Sexual-Attitude Permissiveness PC1	−0.0246	0.2504	[−0.5153, 0.4662]	0.9218
HIV-related social distancing	−3.1570	1.0329	[−5.1814, −1.1325]	0.0022
**Model fit: R^2 = 0.5536; adjusted R^2 = 0.5176; AIC = 255.7768; BIC = 269.0939**				
**Wave-year adjusted, n = 68**				
log GDP	−1.1826	0.2411	[−1.6551, −0.7101]	< 0.001
Net GINI	0.0765	0.0329	[0.0121, 0.1408]	0.0200
Religiosity PC1	−0.1408	0.2050	[−0.5427, 0.2610]	0.4922
Sexual-Attitude Permissiveness PC1	−0.0553	0.2115	[−0.4697, 0.3592]	0.7938
HIV-related social distancing	−3.0705	0.9454	[−4.9234, −1.2176]	0.0012
Centered wave year	0.0419	0.0327	[−0.0222, 0.1060]	0.2004
**Model fit: R^2 = 0.5694; adjusted R^2 = 0.5270; AIC = 255.3355; BIC = 270.8721**				
**2015+ WVS waves, n = 46**				
log GDP	−1.1452	0.2532	[−1.6415, −0.6490]	< 0.001
Net GINI	0.0343	0.0362	[−0.0367, 0.1053]	0.3434
Religiosity PC1	−0.1529	0.2210	[−0.5861, 0.2803]	0.4890
Sexual-Attitude Permissiveness PC1	−0.0821	0.2165	[−0.5064, 0.3422]	0.7046
HIV-related social distancing	−2.6055	1.1576	[−4.8744, −0.3366]	0.0244
**Model fit: R^2 = 0.5373; adjusted R^2 = 0.4794; AIC = 162.5572; BIC = 173.5291**				
**World Bank region fixed effects, n = 68**				
log GDP	−0.4589	0.2196	[−0.8892, −0.0285]	0.0366
Net GINI	0.0310	0.0269	[−0.0217, 0.0837]	0.2496
Religiosity PC1	0.0645	0.1773	[−0.2830, 0.4120]	0.7160
Sexual-Attitude Permissiveness PC1	0.0581	0.2161	[−0.3654, 0.4815]	0.7882
HIV-related social distancing	−0.3643	1.1213	[−2.5619, 1.8334]	0.7453
**Model fit: R^2 = 0.7327; adjusted R^2 = 0.6802; AIC = 232.9082; BIC = 259.5423**				
**Panel B. STI incidence (log)**				
**Predictor**	**b**	**HC3 SE**	**95% CI**	**p**
**Reference (Model 3), n = 68**				
log GDP	−0.5366	0.0947	[−0.7222, −0.3510]	< 0.001
Net GINI	0.0113	0.0129	[−0.0140, 0.0365]	0.3809
Religiosity PC1	−0.1787	0.1090	[−0.3923, 0.0350]	0.1012
Sexual-Attitude Permissiveness PC1	−0.1068	0.1061	[−0.3148, 0.1012]	0.3142
HIV-related social distancing	−1.5610	0.4277	[−2.3993, −0.7226]	< 0.001
**Model fit: R^2 = 0.5310; adjusted R^2 = 0.4932; AIC = 142.5349; BIC = 155.8520**				
**Wave-year adjusted, n = 68**				
log GDP	−0.5167	0.0988	[−0.7104, −0.3231]	< 0.001
Net GINI	0.0108	0.0134	[−0.0155, 0.0371]	0.4215
Religiosity PC1	−0.1644	0.1153	[−0.3905, 0.0616]	0.1540
Sexual-Attitude Permissiveness PC1	−0.1017	0.1075	[−0.3124, 0.1090]	0.3442
HIV-related social distancing	−1.5754	0.4250	[−2.4084, −0.7423]	< 0.001
Centered wave year	−0.0070	0.0179	[−0.0420, 0.0281]	0.6971
**Model fit: R^2 = 0.5334; adjusted R^2 = 0.4875; AIC = 144.1834; BIC = 159.7200**				
**2015+ WVS waves, n = 46**				
log GDP	−0.5194	0.0878	[−0.6914, −0.3473]	< 0.001
Net GINI	−0.0113	0.0137	[−0.0382, 0.0155]	0.4088

Religiosity PC1	−0.1255	0.0936	[−0.3089, 0.0579]	0.1800
Sexual-Attitude Permissiveness PC1	−0.0811	0.0987	[−0.2745, 0.1123]	0.4111
HIV-related social distancing	−1.8457	0.4357	[−2.6997, −0.9918]	< 0.001
**Model fit: R² = 0.6171; adjusted R² = 0.5692; AIC = 74.0446; BIC = 85.0164**				
**World Bank region fixed effects, n = 68**				
log GDP	−0.4060	0.1370	[−0.6745, −0.1375]	0.0030
Net GINI	0.0137	0.0164	[−0.0184, 0.0458]	0.4034
Religiosity PC1	−0.1597	0.1144	[−0.3838, 0.0645]	0.1627
Sexual-Attitude Permissiveness PC1	−0.0731	0.1134	[−0.2953, 0.1491]	0.5193
HIV-related social distancing	−0.8005	0.6064	[−1.9891, 0.3880]	0.1868
**Model fit: R² = 0.6254; adjusted R² = 0.5519; AIC = 139.2455; BIC = 165.8796**				

Values are unstandardized regression coefficients estimated with HC3 heteroskedasticity-consistent covariance matrices. The wave-year model adds a centered WVS survey-year term; the 2015+ model restricts the sample to countries whose selected WVS wave occurred in 2015 or later; and the region fixed-effect model adds six World Bank region indicators. The inverse associations of HIV-related social distancing with both outcomes remained under wave-year adjustment and the 2015+ restriction but were substantially attenuated and no longer statistically significant after region fixed effects were included, indicating a predominantly between-region pattern.

Table S8. Ascertainment sensitivity: adjusting the social-distancing→HIV association for ART coverage

**Specification**	**n**	**Social distancing→HIV (no ART)**	**Social distancing→HIV (+ART)**	**Attenuation**	**ART→HIV**	**Indirect θ (95% CI)**
ART coverage (recent), full sample	55	−3.67 (<0.001)	−3.18 (<0.001)	13%	0.028 (0.003)	−0.49 (−1.38, 0.09)
ART coverage (wave-year), full sample	50	−3.50 (<0.001)	−2.86 (0.001)	18%	0.021 (0.077)	−0.64 (−1.71, 0.26)
ART coverage (recent), 2015+ waves	36	−2.48 (0.062)	−1.64 (0.117)	34%	0.041 (<0.001)	−0.84 (−2.37, 0.91)

ART coverage = percentage of people living with HIV receiving antiretroviral therapy (UNAIDS via World Bank), used as a proxy for the completeness of the HIV detection-and-treatment cascade, although it may also reflect treatment access and survival and should not be interpreted as a direct measure of HIV testing; available for 55 of 68 countries (high-prevalence settings such as the Russian Federation and Ukraine were among those without data). "Recent" uses each country's most recent estimate; "wave-year" matches the WVS survey year (mechanically low for pre-2005 waves, before global ART scale-up). All models adjust for log GDP and GDP-adjusted GINI with HC3 robust standard errors; the exploratory indirect effect is from a 5,000-iteration bootstrap. Social-distancing coefficients attenuate but the inverse association persists in the full sample, and the exploratory indirect effect is not statistically significant. The indirect-effect estimate is exploratory and does not establish causal mediation because the data are cross-sectional and ecological and do not demonstrate temporal ordering or sequential ignorability.

6 Exploratory Indirect-Effect Decomposition (Product-of-Coefficients)

The main-text exploratory indirect-effect decomposition (Table 3) follows a product-of-coefficients approach. For the HIV outcome, the descriptive indirect/total ratio is approximately 98.1%. This ratio is descriptive only and does not represent causal mediation. These estimates describe a product-of-coefficients pattern and do not establish causal mediation because the study is cross-sectional and ecological and does not demonstrate temporal ordering or sequential ignorability. Regression coefficients (Tables S7 and S8) use HC3 robust 95% CIs; the indirect effect is summarized with the Sobel normal-theory 95% CI and the bias-corrected and accelerated (BCa) bootstrap 95% CI. These interval types are not interchangeable. Full decomposition values for both outcomes appear in Table S9 below.

Values are unstandardized coefficients (b) with heteroscedasticity-consistent (HC3) standard errors in parentheses; all models control for log GDP per capita and Net GINI (GDP-adjusted GINI). HIV-related social distancing is a single general-population social-distancing item (unwillingness to have a person with AIDS as a neighbor) and is not a comprehensive stigma index. The indirect effect (a × b)

is accompanied by the Sobel normal-theory 95% CI and a bias-corrected and accelerated (BCa) bootstrap 95% CI (5,000 iterations); these differ from the HC3 coefficient CIs and are not interchangeable. The descriptive indirect/total ratio is descriptive only and does not represent causal mediation, because the design is cross-sectional and ecological and does not establish temporal ordering or sequential ignorability.

Table S9. Decomposition of the association between sexual-attitude permissiveness and epidemiological outcomes through HIV-related social distancing (exploratory, product-of-coefficients)

**Parameter**	**HIV (log prevalence)**				**STI (log incidence)**			
	b	**SE**	**95% CI**	***p***	***b***	**SE**	**95% CI**	***p***
**a path** Sexual-Attitude Permissiveness PC1 → HIV-related social distancing	−0.126	0.035	[−0.195, −0.057]	< 0.001	−0.126	0.035	[−0.195, −0.057]	< 0.001
**b path** HIV-related social distancing → outcome	−3.063	1.018	[−5.058, −1.068]	0.003	−1.255	0.438	[−2.113, −0.397]	0.004
**Total association (c)**	0.393	0.142	[0.115, 0.671]	0.006	0.155	0.089	[−0.019, 0.329]	0.083
**Direct association (c′)**	0.007	0.226	[−0.436, 0.450]	0.974	−0.003	0.093	[−0.185, 0.179]	0.974
**Indirect effect (a × b)**	0.386	0.167	[0.058, 0.714] *Sobel*	0.022	0.158	0.071	[0.020, 0.296] *Sobel*	0.026
**Sobel test**	z = 2.296		—	0.022	z = 2.231		—	0.026
**BCa bootstrap 95% CI** (indirect effect)	—	—	[0.071, 0.790]	0.001_{boot}	—	—	[0.061, 0.307]	0.016_{boot}
**Proportion mediated**	≈ 98.1% (descriptive only)				Not reported (total effect ≈ 0, unstable)			

b = unstandardized coefficient; SE = standard error; CI = confidence interval; PC1 = first principal component of sexual-attitude permissiveness. The indirect effect (a × b) is tested with the Sobel test and with bias-corrected and accelerated (BCa) bootstrap confidence intervals (pboot, 5,000 resamples). For the indirect-effect row the reported interval is the Sobel CI. The descriptive indirect/total ratio is descriptive and is not reported for the STI model because the total effect is near zero and the estimate is unstable. Em dash (—) indicates not applicable.
